# Cytoskeletal control in adult microglia is essential to restore neurodevelopmental synaptic and cognitive deficits

**DOI:** 10.1126/sciadv.adw0128

**Published:** 2025-08-29

**Authors:** Sofie Kessels, Chloë Trippaers, Melanie Mertens, Ibrahim Hamad, Ben Rombaut, Art Janssen, Keerthana Ramanathan, Sam Duwé, Adelaïde M. Gharghani, Rune Theuwis, Amber Delbroek, Tim Vangansewinkel, Lisa Berden, Jolien Beeken, Patrick Vandormael, Suresh Poovathingal, Thomas Voets, Markus Kleinewietfeld, Laurent Nguyen, Jack P. Antel, Luke M. Healy, Sally A. Cowley, Koko Ishizuka, Jean-Michel Rigo, Jelle Hendrix, Tim Vanmierlo, Ilse Dewachter, Yeranddy A. Alpizar, Akira Sawa, Bert Brône

**Affiliations:** ^1^ Biomedical Research Institute, BIOMED, Hasselt University, UHasselt, 3590 Diepenbeek, Belgium.; ^2^ Department of Psychiatry, Johns Hopkins University School of Medicine, Baltimore, MD, USA.; ^3^ Laboratory of Translational Immunomodulation, VIB Center for Inflammation Research (IRC), Hasselt University, 3590 Diepenbeek, Belgium.; ^4^ Department Psychiatry and Neuropsychology, European Graduate School of Neuroscience, Mental Health and Neuroscience Research Institute, Division of Translational Neuroscience, Maastricht University, Maastricht, Netherlands.; ^5^ Dynamic Bioimaging Lab, Advanced Optical Microscopy Center, Biomedical Research Institute, Hasselt University, Hasselt, Belgium.; ^6^ Mechanobiology & Biomaterials Group, CIRMAP, Research Institute for Biosciences, University of Mons, 7000 Mons, Belgium.; ^7^ Radiobiology Unit, Nuclear Medical Applications Institute, Belgian Nuclear Research Centre (SCK CEN), Mol, Belgium.; ^8^ Laboratory of Molecular Regulation of Neurogenesis, GIGA Institute, ULiège, C.H.U. Sart Tilman, Liège, Belgium.; ^9^ KU Leuven, Department of Neurosciences, Leuven Brain Institute, Leuven, Belgium.; ^10^ VIB Center for Brain & Disease Research, Leuven, Belgium.; ^11^ Laboratory of Ion Channel Research and TRP Research Platform Leuven (TRPLe), Department of Cellular and Molecular Medicine, University of Leuven, Leuven, Belgium.; ^12^ Department of Neurology and Neurosurgery, Montreal Neurological Institute, McGill University, Québec, Canada.; ^13^ Department of Neuroscience, Johns Hopkins University School of Medicine, Baltimore, MD, USA.; ^14^ Department of Pharmacology, Johns Hopkins University School of Medicine, Baltimore, MD, USA.; ^15^ Department of Biomedical Engineering, Johns Hopkins University School of Medicine, Baltimore, MD, USA.; ^16^ Department of Genetic Medicine, Johns Hopkins University School of Medicine, Baltimore, MD, USA.; ^17^ Department of Mental Health, Johns Hopkins Bloomberg School of Public Health, Baltimore, MD, USA.

## Abstract

Synaptic dysfunction is a hallmark of neurodevelopmental disorders (NDDs), often linked to genes involved in cytoskeletal regulation. While the role of these genes has been extensively studied in neurons, microglial functions such as phagocytosis are also dependent on cytoskeletal dynamics. We demonstrate that disturbance of actin cytoskeletal regulation in microglia, modeled by genetically impairing the scaffold protein Disrupted-in-Schizophrenia 1 (DISC1), which integrates actin-binding proteins, causes a shift in actin regulatory balance favoring filopodial versus lamellipodial actin organization. The resulting microglia-specific dysregulation of actin dynamics leads to excessive uptake of synaptic proteins. Genetically engineered DISC1-deficient mice show diminished hippocampal excitatory transmission and associated spatial memory deficits. Reintroducing wild-type microglia-like cells via bone marrow transplantation in adult DISC1-deficient mice restores the synaptic function of neurons and rescues cognitive performance. These findings reveal a pivotal role for microglial actin cytoskeletal remodeling in preserving synaptic integrity and cognitive health. Targeting microglial cytoskeletal dynamics may effectively address cognitive impairments associated with NDDs, even in adulthood.

## Teaser

Microglial actin cytoskeletal dysregulation links excessive synaptic elimination to memory loss in neurodevelopmental conditions.

## INTRODUCTION

Atypical brain development and synaptic dysfunction are hallmark features of neurodevelopmental disorders (NDDs), leading to substantial disruptions in adult brain connectivity that manifest as cognitive impairments, behavioral abnormalities, and neuropsychiatric symptoms (*1*, *2*). The genetic risk factors underlying NDDs often involve mutations in genes encoding cytoskeletal proteins. These proteins have predominantly been studied in neurons, where they are essential for maintaining structural integrity, facilitating intracellular transport, and supporting synaptic function (*3*–*16*). However, their role extends beyond neurons, in particular, playing a critical role in microglia—the brain’s resident immune cells and phagocytes.

Microglia are increasingly recognized as key regulators of neurodevelopment, actively participating in processes such as neuronal survival, synapse formation, and synaptic elimination, which are essential for establishing proper neural circuits (*9*, *17*). Microglia efficiently survey their environment, migrate to sites of injury, and engulf and degrade cellular debris and excess synapses through phagocytosis (*18*, *19*). Despite the critical importance of these processes, the molecular mechanisms that govern cytoskeletal dynamics in microglia, particularly in the context of NDDs, remain poorly understood.

In this study, we seek to address this gap by investigating the role of cytoskeletal dynamics in microglia, with a particular focus on their involvement in synapse elimination and memory maintenance—two processes crucial for proper functioning of the adult brain. Scaffold proteins play a key role in physically connecting cytoskeletal molecules and coordinating their function. Disrupted-in-Schizophrenia 1 (DISC1) is one of the representative scaffold proteins that interact both with actin- and microtubule-cytoskeletal proteins, and with proteins involved in neuronal and synaptic organization and function, many of which have been implicated in NDDs such as lissencephaly-1 (LIS1), nudE neurodevelopment protein 1 like 1 (NDEL1), and kalirin-7 (Kal7) (*20*–*22*).

To investigate the involvement of the microglial cytoskeleton in brain development and cognitive behavior, we used a mouse model deficient for DISC1, i.e., the *Disc1* locus impairment (*Disc1* LI) model, which is equivalent to a knockout. By elucidating the role of cytoskeletal dynamics specifically in microglia, we shed light on the molecular mechanisms underlying microglial involvement in NDDs. Our findings reveal how disruptions in cytoskeleton rearrangements in microglia contribute to synaptic abnormalities and cognitive deficits, and show the potential to rescue memory deficits and synapse function by restoring actin-dependent control of phagocytosis in adult microglia.

## RESULTS

### DISC1 skews the microglial actin cytoskeleton toward a lamellipodia-favoring morphology

**Fig. 1.**
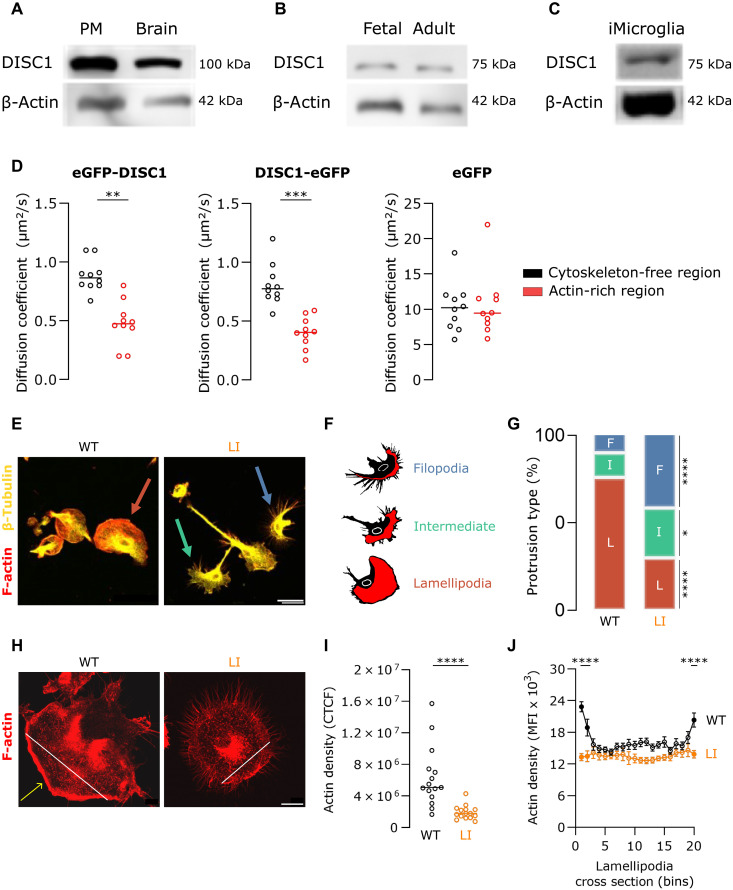
The actin cytoskeleton in microglia is disrupted by DISC1 deficiency. DISC1 levels in (**A**) mouse primary microglia and whole brain lysates, (**B**) fetal and adult human microglia, and (**C**) human iMicroglia. PM, primary microglia. (**D**) BV2 cells, comagnetofected with N- or C-terminal eGFP-tagged DISC1 and mCherry-LifeAct, were incubated with SPY650-tubulin to determine the diffusion coefficient of eGFP-tagged DISC1 in cytoskeleton-free cellular regions and actin-rich regions. *n* = 10 per condition. Data points represent individual microglia. Horizontal bars indicate the median. Shapiro-Wilk test followed by the two-tailed Mann-Whitney *U* test. (**E**) Representative F-actin (red) and β-tubulin (yellow)–stained *Disc1^WT/WT^* and *Disc1^LI/LI^* primary microglia. Scale bar, 25 μm. (**F**) Schematic overview of three categories of microglial morphologies based on actin-rich protrusions (lamellipodia area in red). Colored arrows in (E) indicate an example per protrusion type: cells with large round lamellipodia (red), small spikey filopodia (blue), and intermediate shapes (green). (**G**) Proportion of different protrusion-based morphologies. Fisher’s exact test. F, filopodia; I, intermediate; L, lamellipodia. (**H**) Representative phalloidin staining in *Disc1^WT/WT^* and *Disc1^LI/LI^* primary microglia. The yellow arrow indicates the actin cortex in *Disc1^WT/WT^* cells. White lines indicate cross sections of lamellipodia used for analysis in (J). Scale bar, 10 μm. (**I**) Total F-actin density was quantified and normalized using the corrected total cell fluorescence (CTCF) method. *n* = 15 per genotype. Data points represent individual microglia. Horizontal bars indicate the median. Shapiro-Wilk test followed by the two-tailed Mann-Whitney *U* test. (**J**) F-actin density was measured over binarized cross sections of primary microglia lamellipodia. *n* = 15 per genotype. Data are reported as mean ± SEM. Shapiro-Wilk test followed by two-way analysis of variance (ANOVA) with Šidák’s multiple comparisons test. Filled data points indicate significant differences (*P* < 0.001) from the equidistant value in WT. *P < 0.05, **P < 0.01, ***P < 0.001, and ****P < 0.0001

To validate DISC1 as a regulator of the actin cytoskeleton in microglia, we confirmed its expression in primary mouse microglia, fetal and adult human microglia, and human induced pluripotent stem cells (iPSC)-derived microglia (iMicroglia) (Fig. 1, A to C). DISC1’s interaction with actin and/or actin-binding proteins was studied by cotransfecting murine microglial BV2 cells with plasmids expressing DISC1 fused to enhanced green fluorescent protein (eGFP) at either the N or C terminus, along with an mCherry-LifeAct plasmid for visualization of filamentous actin (F-actin). Raster image correlation spectroscopy (RICS) showed that the diffusion coefficient of eGFP-tagged DISC1 was significantly reduced in actin-rich regions compared to cytoskeleton-free regions, regardless of whether eGFP was fused to the N terminus (eGFP-DISC1) or C terminus (DISC1-eGFP) (Fig. 1D). This reduced DISC1 diffusion in actin-rich regions suggests a direct or indirect interaction between DISC1 and microglial F-actin.

To characterize the impact of DISC1 on microglial actin organization, we analyzed their morphological features based on actin-rich protrusions, i.e.*,* lamellipodia or filopodia. Lamellipodia appear as broad, sheetlike protrusions supported by a branched F-actin network, while filopodia are slender cytoplasmic projections containing linear F-actin that extend beyond the leading edge of lamellipodia (*23*). Primary cultured microglia from *Disc1* wild-type (^WT/WT^) and *Disc1* LI (^LI/LI^) mice were categorized on the basis of their main protrusion type: lamellipodia or filopodia (Fig. 1, E and F). In case the protrusions did not exhibit either morphology, the microglia were classified as “intermediate” (Fig. 1F). Quantification revealed that 76% of the *Disc1^WT/WT^* microglia predominantly showed a lamellipodial morphology (76%), with fewer filopodial (10%) and intermediate shapes (14%). In contrast, *Disc1^LI/LI^* microglia exhibited a marked shift in protrusion type, with a higher proportion of filopodia-rich microglia (41%) and a more balanced distribution of lamellipodial (30%) and intermediate morphologies (28%) (Fig. 1G). Super-resolution imaging further revealed a reduced F-actin density in *Disc1*^LI/LI^ microglia compared to *Disc1^WT/WT^* microglia (Fig. 1, H and I). In *Disc1^WT/WT^* cells, F-actin is concentrated in a dense, cross-linked meshwork at the cell periphery, consistent with the presence of mature lamellipodia (Fig. 1H, indicated by the yellow arrow). In contrast, *Disc1^LI/LI^* microglia exhibited a sparser F-actin distribution, with a predominance of linear filament arrangements characteristic of filopodia. Quantitative analysis confirmed these observations, revealing uniform F-actin distribution across binarized cross sections of *Disc1^LI/LI^* lamellipodia and significantly higher F-actin densities at the cell borders of *Disc1^WT/WT^* lamellipodia (Fig. 1, H and J). These results underscore the cell-intrinsic regulatory role of DISC1 in microglial actin organization by promoting lamellipodia formation. Loss of DISC1 induces a shift toward a filopodia-dominated morphology, potentially altering microglial functions.

### Microglial DISC1 tunes the balance between lamellipodial and filopodial motility

**Fig. 2.**
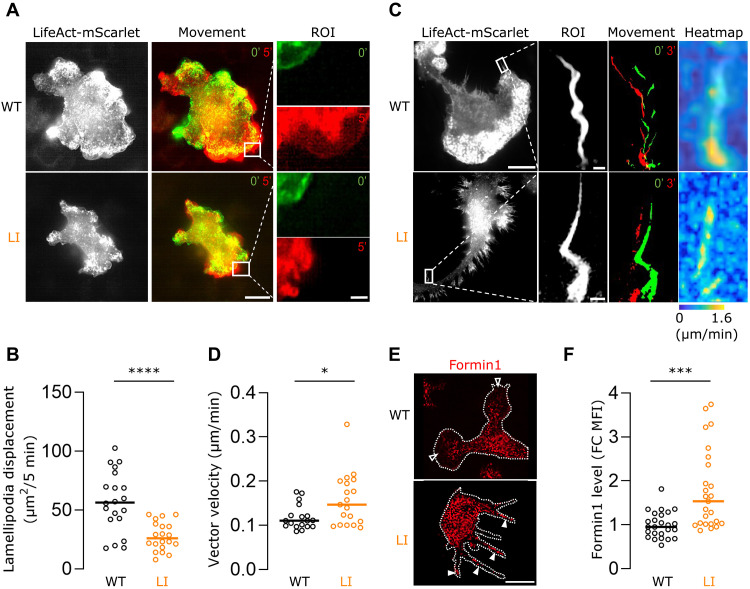
DISC1 differentially regulates F-actin rearrangements in microglial filopodia and lamellipodia. (**A**) Representative SIM images of F-actin structures observed in *Disc1^WT/WT^* and *Disc1^LI/LI^* primary microglia transduced with LifeAct-mScarlet lentivirus. Scale bar, 10 μm. Green and red colors represent the displacement between two consecutive images taken 5 min apart, with one region of interest (ROI) shown in the right panel. Scale bar, 1 μm. (**B**) Total lamellipodia displacement of *Disc1^WT/WT^* and *Disc1^LI/LI^* primary microglia. (**C**) Representative SIM images of F-actin structures observed in *Disc1^WT/WT^* and *Disc1^LI/LI^* primary microglia transduced with LifeAct-mScarlet lentivirus. Scale bar, 10 μm. Green and red colors represent the displacement between two consecutive images taken 3 min apart, with one ROI shown in the second panel. Scale bar, 1 μm. PIVlab-generated heat maps of particle vector velocity calculation are shown in the right panel. The color-coded bar indicates the range of vector velocity magnitude. (**D**) Mean vector velocity values of all interrogation areas of the representative *Disc1^WT/WT^* and *Disc1^LI/LI^* primary microglia shown in (C). (**E**) Representative images of immunocytochemical staining of Formin1 in *Disc1^WT/WT^* and *Disc1^LI/LI^* primary microglia. Scale bar, 10 μm. (**F**) Fold change (FC) quantification of Formin1 mean fluorescence intensity (MFI) in *Disc1^WT/WT^* and *Disc1^LI/LI^* primary microglia. Data points represent individual microglia. [(B) and (D)] Data points represent individually analyzed ROIs of different images (*n* = 20 regions per condition, from three independent experiments). Shapiro-Wilk test followed by (B) two-tailed unpaired *t* test or [(D) and (F)] two-tailed Mann-Whitney *U* test. *P < 0.05, ***P < 0.001, and ****P < 0.0001

To further dissect how DISC1 regulates cytoskeletal behavior beyond structural morphology, we next assessed F-actin rearrangements in primary microglia using live-cell structured illumination microscopy (SIM). For this, we transduced *Disc1^WT/WT^* and *Disc1^LI/LI^* primary microglia with LifeAct-mScarlet lentivirus to visualize F-actin dynamics in real time. In lamellipodia, actin rearrangements were monitored by tracking F-actin displacement over time (5-min interval) (Fig. 2A). *Disc1^LI/LI^* microglia exhibited significantly reduced lamellipodial displacement compared to *Disc1^WT/WT^* cells (Fig. 2B), indicating impaired remodeling of the branched F-actin network within these structures. In addition, filopodia dynamics were analyzed in LifeAct-mScarlet–transduced primary microglia by recording consecutive SIM time-lapse images and applying particle image velocimetry (PIV) to calculate local vector velocities within defined interrogation windows (Fig. 2C). Vector quantification revealed significantly increased displacement of linear F-actin in filopodia of *Disc1^LI/LI^* microglia relative to *Disc1^WT/WT^* filopodia (Fig. 2D). As Formin1 is a key actin-nucleating protein and regulator of linear actin polymerization (*24*), we next examined the abundance and subcellular localization of Formin1 using immunocytochemistry in *Disc1^WT/WT^* and *Disc1^LI/LI^* primary microglia (Fig. 2E). Formin1 levels were markedly elevated in *Disc1^LI/LI^* cells compared to *Disc1^WT/WT^* counterparts (Fig. 2F), and it is predominantly localized to filopodial extensions (full arrowheads in Fig. 2E) rather than lamellipodia (empty arrowheads in Fig. 2E). This spatial enrichment aligns with the established role of Formin1 in driving linear F-actin polymerization and promoting filopodia formation and motility. Together, these findings suggest that DISC1 ensures lamellipodia formation in microglia and that DISC1 deficiency in microglia drives the formation of filopodial extensions by enhancing F-actin rearrangement into parallel filaments at the cost of the lamellipodial branched actin network structure.

### Actin dysregulation and hyperphagocytosis characterize the transcriptome of DISC1-deficient microglia

**Fig. 3.**
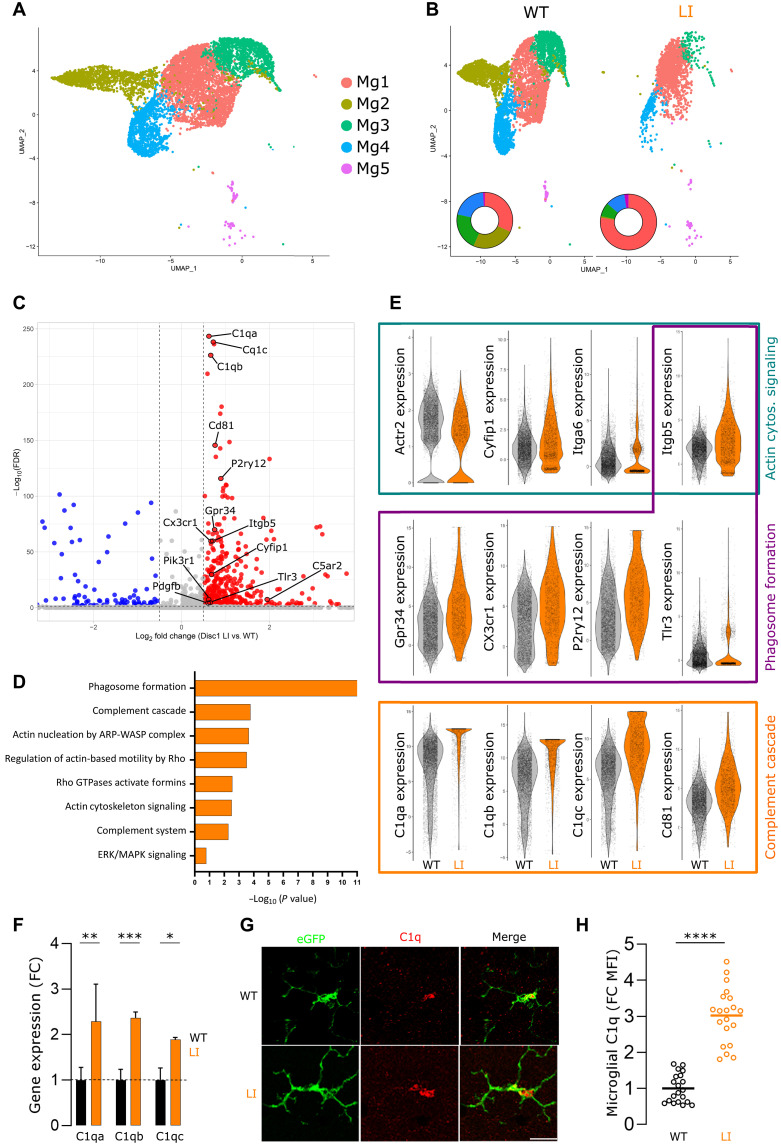
Disruption of actin cytoskeleton signaling impairs phagocytic and motility capacity in *Disc1* LI microglia. (**A**) Uniform manifold approximation and projection (UMAP) analysis of 6072 *Disc1^WT/WT^* and 2040 *Disc1^LI/LI^* microglia from 23-week-old mice grouped into five clusters. Each dot represents a cell, color-coded by cluster affiliation. (**B**) UMAP illustrates the distribution of microglia among *Disc1^WT/WT^* and *Disc1^LI/LI^* genotypes. A differential abundance analysis shows the percentage of cells in each cluster for both genotypes, revealing cluster Mg1 as the most abundant in *Disc1^LI/LI^* mice, while they lack cluster Mg2. (**C**) Volcano plot depicting differentially expressed genes (DEGs) in *Disc1^LI/LI^* versus *Disc1^WT/WT^* microglia. The *y* axis indicates the statistical significance [false discovery rate (FDR)–adjusted *P* value], with up-regulated genes highlighted in red and down-regulated genes in blue. Genes surpassing the significance threshold (adjusted *P* < 0.05) are considered statistically significant. (**D**) Ingenuity Pathway Analysis (IPA) prediction of selected canonical pathways that are significantly deregulated in *Disc1^LI/LI^* microglia. ERK, extracellular signal–regulated kinase; MAPK, mitogen-activated protein kinase. (**E**) Gene expression (log_10_) for distinct marker genes related to phagosome formation, actin cytoskeleton signaling, and complement cascade pathways was compared between *Disc1^WT/WT^* and *Disc1^LI/LI^* microglia. Data points represent individual cells. cytos., cytoskeleton. (**F**) Quantitative polymerase chain reaction (qPCR) analysis of complement genes *C1qa*, *C1qb*, and *C1qc* in *Disc1^WT/WT^* and *Disc1^LI/LI^* microglia isolated from adult mice. Fold change (FC) data are represented as mean ± SEM from three independent experiments. Two-way ANOVA with Tukey’s multiple comparisons test. (**G**) Representative images of a C1q immunostaining of hippocampal tissue slices of adult *Disc1^WT/WT^ Cx3cr1^eGFP/+^* and *Disc1^LI/LI^ Cx3cr1^eGFP/+^* mice. Scale bar, 10 μm. (**H**) FC quantification of C1q MFI in eGFP-positive microglia in situ. Shapiro-Wilk test followed by an unpaired two-tailed *t* test. *P < 0.05, **P < 0.01, ***P < 0.001, and ****P < 0.0001.

Our findings highlight that DISC1 deficiency results in differential actin rearrangements favoring filopodial cytoskeletal structures and suggest that distinct regulatory mechanisms of actin organization and dynamics are in place. It is well established that lamellipodia formation depends on branched actin networks regulated by the Rac-WAVE-ARP2/3 pathway, whereas filopodia rely on linear actin filaments governed by Rho family guanosine triphosphatases (Rho, Cdc42) and formin activity (*24*, *25*). To address the impact of DISC1 deficiency on actin regulatory mechanisms and associated signaling pathways in microglia, we performed a comparative gene expression analysis between *Disc1^LI/LI^* and *Disc1^WT/WT^* microglia using single-cell RNA sequencing (scRNA-seq). The analysis identified five unique microglia clusters representing different functional states (Fig. 3, A and B, and fig. S1). *Disc1^LI/LI^* microglia exhibited significantly reduced transcriptional complexity, with a decrease in the Mg2, Mg3, and Mg4 clusters being compensated by a proportional increase in the Mg1 cluster (Fig. 3B).

Differential gene expression analysis revealed, besides an expected disruption in actin cytoskeleton signaling, that the most significant differentially expressed genes (DEGs) in *Disc1^LI/LI^* microglia were related to the complement cascade, particularly those involved in synaptic elimination (e.g., *C1q*, *Cd81*, and *C5ar2*) (Fig. 3, C to E, and fig. S2) (*26*). We validated the upregulation of *C1qa*, *C1qb*, and *C1qc* in *Disc1^LI/LI^* microglia using quantitative polymerase chain reaction (qPCR) (Fig. 3F), confirming increased transcript levels of these C1q subcomponents in isolated cells. In situ immunostaining further demonstrated elevated C1q levels in microglia within the brain parenchyma (Fig. 3, G and H), supporting enhanced complement activity in microglia at both the transcript and protein level. Ingenuity Pathway Analysis (IPA) identified signaling pathways with differential enrichment in adult *Disc1^LI/LI^* microglia, confirming that, in addition to actin cytoskeleton signaling, RNA transcripts related to phagosome formation and the complement cascade were strongly increased (Fig. 3D). Among the DEGs related to actin cytoskeleton dynamics, we observed a significant reduction in the expression of *Actr2* and *Actr3*, encoding ARP2 and ARP3, in *Disc1^LI/LI^* microglia. The ARP2/3 complex is essential for reorganizing the actin cytoskeleton into a dense, cross-linked network of F-actin at the edges of microglial lamellipodia (*27*). Its down-regulation in *Disc1^LI/LI^* microglia correlates with impaired lamellipodia formation (Figs. 1, E to G, and 2, A and B), disrupted actin cortex integrity (Fig. 1, H to J), and decreased F-actin motility within lamellipodia (Fig. 2, A and B). In addition, *Disc1^LI/LI^* microglia show increased *Cyfip1* expression (Fig. 3, C and E), which is an NDD risk gene involved in both phagocytosis and the ARP2/3 pathway (*28*). In contrast, several formin-associated genes, including *Fmn1*, *Rhoj*, *Diaph2/3*, *and Arhgef12*, were upregulated in *Disc1^LI/LI^* cells. Formins promote the nucleation and elongation of linearized F-actin filaments and play key roles in filopodia formation. Consistently, we observed increased filopodia formation and enhanced F-actin motility in these structures in *Disc1^LI/LI^* microglia (Fig. 1, E to G). These findings suggest a DISC1-dependent shift in actin remodeling pathways in microglia, in which DISC1 deficiency induces a shift from ARP2/3-dependent lamellipodia to formin-driven filopodia.

Gene expression patterns in the microglia population designated the Mg1 cluster as highly phagocytic microglia due to their increased expression of genes associated with the complement cascade and phagosome formation (fig. S2). The Mg1 cluster is also enriched in homeostatic microglia markers such as *P2ry12* and *Cx3cr1*, similar to the Mg3 cluster (fig. S2) (*29*). Notably, one specific cluster, characterized by distinct *Cd55* expression, was completely absent in *Disc1^LI/LI^* microglia (Fig. 2, A and B, and figs. S1 and S3; olive Mg2 cluster). This cluster, which comprises ~25% of the microglia in WT controls (see pie chart in the inset of Fig. 2B), appears to function as an antiphagocytic population, given CD55’s role in inhibiting the complement cascade (*30*, *31*). These results suggest that DISC1 and actin cytoskeleton rearrangements modulate a balance between pro- and antiphagocytic states in the homeostatic microglia population.

### Cytoskeleton dysregulation by DISC1 deficiency alters microglial surveillance and phagocytosis

**Fig. 4.**
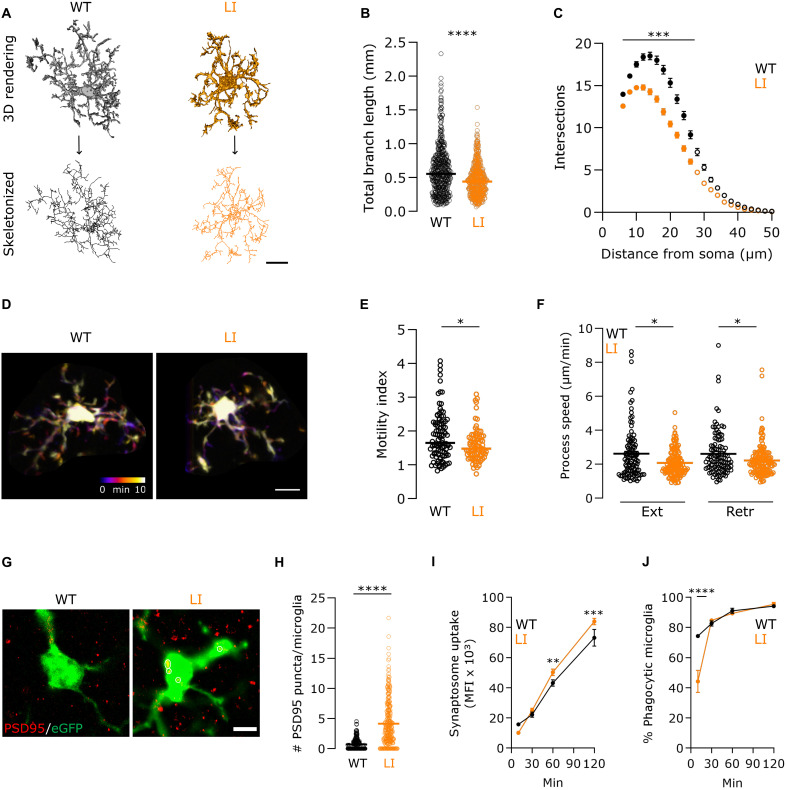
Cytoskeletal dysregulation caused by DISC1 deficiency alters microglial morphology, surveillance, and phagocytosis. (**A**) Representative three-dimensional (3D) renders and skeletonized in situ *Disc1^WT/WT^ Cx3cr1^eGFP/+^* and *Disc1^LI/LI^ Cx3cr1^eGFP/+^* postnatal day 21 (P21) microglia. Scale bar, 10 μm. (**B**) Total branch length (*P* < 0.0001), and (**C**) the number of Sholl analysis process intersections (*P* < 0.001) of in situ *Disc1^WT/WT^* (456 cells from six animals) and *Disc1^LI/LI^* (447 cells from six animals) microglia. (**D**) Representative two-photon microscopy images of P21 *Disc1^WT/WT^ Cx3cr1^eGFP/+^* and *Disc1^LI/LI^ Cx3cr1^eGFP/+^* microglia. Images taken 1 min apart are overlaid with different colors. Scale bar, 10 μm. (**E**) Motility index of *Disc1^WT/WT^ Cx3cr1^eGFP/+^* (*n* = 105) and *Disc1^LI/LI^ Cx3cr1^eGFP/+^* (*n* = 86) microglia in acute brain slices. (**F**) The speed of extension (Ext) (*P* = 0.0215; *n* ≥ 104 per genotype) and retraction (Retr) (*P* = 0.0203; *n* ≥ 141 per genotype) events was quantified from at least five independent slices per mouse. (**G**) Representative images of postsynaptic density 95 (PSD95; red) immunoreactive puncta in dorsal cornu ammonis 1 (CA1) P21 microglia. Circles indicate overlap. Scale bar, 5 μm. (**H**) Quantitative analysis of (G) indicates increased uptake of synaptic material by *Disc1^LI/LI^* microglia (*P* = 0.0074) (*n* = 300 cells from five mice). (**I**) Flow cytometry–based 1,1′-dioctadecyl-3,3,3′,3′- tetramethylindocarbocyanine perchlorate (DiI)–labeled synaptosome uptake in primary microglia measured by the MFI of phagocytic cells. (**J**) The percentage of phagocytic microglia. *n* = 3; repeated-measures (RM) two-way ANOVA with multiple comparisons. Data are represented as mean ± SD. [(B), (E), (F), and (H)] Data points represent individual microglia. Horizontal bars indicate the median. Shapiro-Wilk test followed by the two-tailed Mann-Whitney *U* test. (C) Filled data points indicate significant differences (*P* < 0.001; multiple two-tailed Mann-Whitney *U* tests) from the equidistant value in WT. Data are represented as mean ± SEM. *P < 0.05, **P < 0.01, ***P < 0.001, and ****P < 0.0001.

As actin rearrangements are disrupted by DISC1 deficiency, an aberrant microglial morphology is expected in *Disc1^LI/LI^* mice. Sholl analysis was performed on microglia in perfusion-fixed brain slices from 21-day-old *Disc1^WT/WT^ Cx3cr1^eGFP/+^* and *Disc1^LI/LI^ Cx3cr1^eGFP/+^* littermates, a developmental stage when microglia typically display a highly ramified morphology. The analysis revealed a significant reduction in total branch length and morphological complexity in *Disc1^LI/LI^* microglia compared to their WT counterparts (Fig. 4, A to C), similar to observations in individuals with NDDs, such as schizophrenia (*32*).

To study the functional consequences of altered actin rearrangement in DISC1-deficient microglia, we investigated the dynamics of microglial processes, characterized by constant extensions and retractions (*33*, *34*). Surveillance capabilities of microglia were assessed using two-photon live imaging in acute brain slices from postnatal day 21 (P21) *Disc1^WT/WT^ Cx3cr1^+/eGFP^* and *Disc1^LI/LI^ Cx3cr1^+/eGFP^* littermates. The dynamic nature of microglia in both *Disc1^WT/WT^* and *Disc1^LI/LI^* mice was evident, with processes continuously scanning the environment, as depicted in the overlaid consecutive images over a 10-min period in Fig. 4D. However, *Disc1^LI/LI^* microglia exhibited a decreased motility index, i.e., surveyed brain area normalized to cell size, compared to WT controls (Fig. 4E). Manual tracking of motile processes further revealed decreased average extension and retraction speeds in *Disc1^LI/LI^* microglia (Fig. 4F). These findings were corroborated by in vitro assessments, which also demonstrated decreased motility in *Disc1^LI/LI^* microglia relative to WT cells (fig. S4), confirming the cell-intrinsic regulation of DISC1 on microglial actin rearrangements and cell motility.

Given the transcriptomic and C1q immunohistochemical evidence indicative of a prophagocytic state in *Disc1^LI/LI^* microglia (Fig. 3), synaptic elimination was investigated at P21—a critical developmental stage for synaptic elimination in the hippocampus. DISC1 deficiency led to a significant increase in the uptake of postsynaptic material during this critical developmental window (Fig. 4, G and H). To determine whether this heightened phagocytic capacity was intrinsic to the *Disc1^LI/LI^* microglia or influenced by the impaired environment, we performed a synaptosome phagocytosis assay on primary cultured *Disc1^LI/LI^* microglia. The uptake of synaptic material was increased after 1 hour (Fig. 4I), yet a significant delay was encountered in the percentage of phagocytic *Disc1^LI/LI^* microglia relative to WT cells (Fig. 4J). These in vitro data indicate that the increased synaptic phagocytosis observed in *Disc1^LI/LI^* microglia is at least partially microglia intrinsic.

### WT microglia restore cognitive and behavioral deficits in *Disc1^LI/LI^* mice

**Fig. 5.**
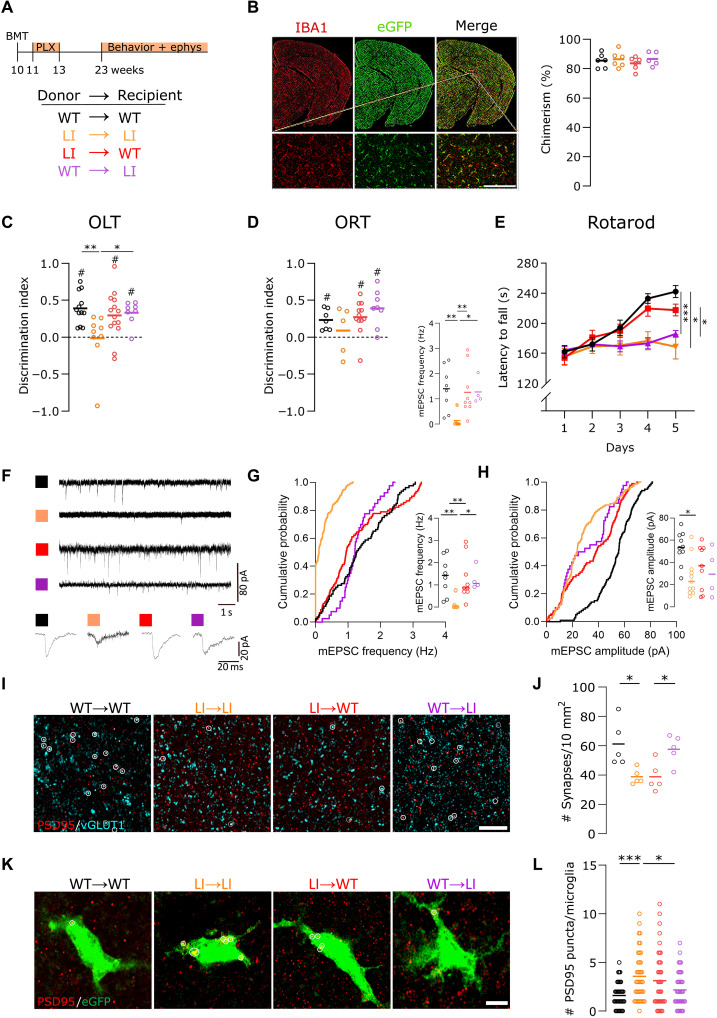
WT microglia-like cells rescue cognitive and synaptic deficits through decreased synaptic elimination in the *Disc1^LI/LI^* hippocampus. (**A**) Experimental setup. Ephys, electrophysiology. (**B**) Representative brain section 13 weeks post-BMT shows that most IBA1-positive microglia-like cells are donor-derived (eGFP^+^). Scale bar, 150 μm. One-way ANOVA with Tukey’s test. (**C**) In the object location test (OLT), LI → LI mice showed a significantly lower discrimination index than WT → WT (*P* = 0.0069) and WT → LI (*P* = 0.0231). (**D**) No significant differences were observed in the object recognition test (ORT). #, significantly different from zero, one-sample *t* test. (**E**) Latency to fall from the rotarod is dependent on recipient genotype (two-way RM ANOVA with Šidák’s test). (**F**) Representative recordings of mEPSCs with an enlarged view of a single synaptic event. (**G**) Cumulative probabilities (Cum. Prob.) of the mEPSC frequencies are plotted. Inset: The corresponding quantitative values of the frequency of mEPSCs per cell. LI → LI versus WT → WT (Cum. Prob. *P* = 0.0001; inset *P* = 0.0027) and versus WT → LI (Cum. Prob. *P* = 0.0048; inset *P* = 0.0440; *n* = 4 to 11 cells). (**H**) Cumulative probabilities and quantification (in inset) of the amplitude of mEPSCs show a significant reduction in the LI → LI mice compared to WT → WT mice (Cum. Prob. *P* > 0.9999; inset *P* = 0.0062) but no significant differences among other groups (*n* = 4 to 11 cells). (**I** and **J**) Vesicular glutamate transporter (vGLUT)1/PSD95 staining in the dorsal hippocampal CA1 region reveals fewer synaptic colocalizations (white circles) in LI → LI (*P* = 0.0223) and LI → WT (*P* = 0.0224) versus WT → WT. Scale bar, 5 μm. (**K** and **L**) Representative images of PSD95^+^ puncta engulfed by dorsal CA1 eGFP^+^ microglia. Scale bar, 5 μm. Quantification shows increased engulfment by LI → LI and LI → WT donor cells (*n* = 40 cells from five mice). [(C) and (D)] Kruskal-Wallis with Dunn’s test. [(G), (H), (J), and (L)] One-way ANOVA with Tukey’s test. *P < 0.05, **P < 0.01, and ***P < 0.001.

The crucial role of microglia in synaptic modulation and elimination is particularly relevant to cognitive phenotypes. Given the complement-enriched transcriptomic profile, increased C1q levels, and increased phagocytic capacity in actin-impaired *Disc1^LI/LI^* microglia (Figs. 3 and 4, G to J), we hypothesized that deficits in spatial memory, motor skill learning, and repetitive behavior characteristic of full *Disc1^LI/LI^* mice (fig. S5, A to O) are mediated by DISC1 deficiency in microglia specifically. In the absence of a conditional *Disc1^LI/LI^* model, we devised a grafting approach in adult mice consisting of full-body irradiation, transplantation of bone marrow obtained from *Cx3cr1^eGFP/+^* donor mice, and a 2-week regimen of PLX5622 (Fig. 5A). This combination of bone marrow transplantation (BMT) and PLX5622 treatment markedly increases the repopulation of the brain with microglia-like cells arising from the transplanted bone marrow (*35*). Our methodology resulted in robust chimerism rates, with engrafted cells exceeding 85% of the microglial population and resident microglia accounting for 15% in all experimental groups, as evidenced by double eGFP/ionized calcium-binding adaptor molecule (IBA)1 expression of bone marrow–derived microglia-like cells. Our BMT strategy allowed us to both disrupt and restore DISC1 expression specifically in microglia-like cells, in any given background (Fig. 5B), resulting in the following experimental groups: WT → WT (control; black), LI → LI (control; orange), LI → WT (microglia-specific *Disc1* LI model; red), and WT → LI (rescue model; purple).

Behavioral tests previously revealing significant differences between full *Disc1^WT/WT^* and full *Disc1^LI/LI^* mice (fig. S5, A to O) were repeated in chimeric mice across the four transplantation groups. Impaired spatial memory, measured using the object location test (OLT), was confirmed in *Disc1^LI/LI^* mice receiving *Disc1^LI/LI^* bone marrow (LI → LI) when compared to WT → WT mice (Fig. 5C). Notably, *Disc1^WT/WT^* mice receiving *Disc1^LI/LI^* bone marrow (LI → WT) exhibited no significant differences compared to the WT → WT group, indicating that the memory impairment in full *Disc1^LI/LI^* mice could not be induced by repopulating *Disc1^LI/LI^* microglia-like cells in adult *Disc1^WT/WT^* recipients (Fig. 5C). Transplanting *Disc1^WT/WT^* bone marrow in *Disc1^LI/LI^* mice (WT → LI) restored the discrimination index to control levels (WT → WT), indicating a rescue of the memory capacity in the WT → LI group when compared to LI → LI mice. A similar trend was observed in the object recognition test (ORT) (Fig. 5D). Mouse behavior in the rotarod test, water splash test, and forced swim test was neither affected nor rescued by transplanting *Disc1^LI/LI^* bone marrow in *Disc1^WT/WT^* mice (LI → WT) and transplanting *Disc1^WT/WT^* bone marrow in *Disc1^LI/LI^* mice (WT → LI), respectively (Fig. 5E and fig. S5, P and Q). The marble burying test did not yield significant differences among the four groups (fig. S5R). However, the impaired nestlet shredding behavior of *Disc1^LI/LI^* mice was rescued by *Disc1^WT/WT^* BMT (WT → LI) (fig. S5S).

In conclusion, repopulating the brain of *Disc1^LI/LI^* recipient mice with adult *Disc1^WT/WT^* microglia-like cells restores cognitive function in the OLT. These data suggest that cytoskeletal disruptions in adult microglia induced by DISC1 deficiency significantly contribute to cognitive symptoms in NDDs.

### WT microglia rescue synaptic function and number in *Disc1^LI/LI^* mice

Because the dorsal hippocampus mediates cognitive processes, such as memory, navigation, and exploration, we focused on the dorsal cornu ammonis 1 (CA1) region of the hippocampus to understand how transplantation with WT bone marrow mitigates the spatial memory deficits of *Disc1^LI/LI^* mice. To assess the impact of microglial DISC1 deficiency on synaptic transmission in CA1 pyramidal neurons, we used whole-cell patch-clamp recordings in acute brain slices of the four chimeric groups after they underwent the battery of behavioral tests (Fig. 5A). The LI → LI glutamatergic neurons exhibited a decreased frequency of miniature excitatory postsynaptic currents (mEPSCs; Fig. 5, F and G) and spontaneous EPSCs (sEPSCs; fig. S6B) compared to the WT → WT mice, suggesting a reduction in excitatory connectivity and a lower number of synapses on CA1 pyramidal neurons. The amplitude of sEPSCs remained largely unchanged (fig. S6C), and the mEPSCs were significantly different in amplitude in the WT → WT versus LI → LI condition (Fig. 5H). These changes in excitatory synaptic transmission were consistent with those observed in the full *Disc1^LI/LI^* model, confirming that they were not artifacts of the BMT procedure (fig. S6, D to G). The LI → WT and WT → LI mice demonstrated frequencies of sEPSCs and mEPSCs similar to those of WT → WT mice, which correlated with their preserved ability to remember spatial information (Fig. 5, F and G, and fig. S6B). Although we cannot exclude a contribution of disrupted synaptic facilitation, our data suggest that the spatial memory deficits in the LI → LI group are associated with a reduced number of synapses on dorsal CA1 neurons.

The functional deficit in synaptic connectivity was validated by a colocalization staining for presynaptic (vGLUT1) and postsynaptic density 95 (PSD95) proteins to measure synapse numbers in the dorsal CA1 region of the hippocampus. As expected, the LI → LI group exhibited a significant reduction in excitatory glutamatergic synapses compared to the WT → WT mice (Fig. 5, I and J). Given the absence of spatial memory deficits, we expected the synaptic density levels in the WT → WT group and experimental groups (LI → WT and WT → LI) to be comparable. However, the LI → WT group exhibited synaptic densities akin to those observed in the memory-impaired LI → LI group. This pattern persisted when examining synaptic engulfment, where puncta of postsynaptic material (PSD95) were quantified in donor bone marrow–derived cells in the hippocampal CA1 region (Fig. 5, K and L). The LI → WT mice displayed postsynaptic material uptake by donor cells comparable to that of the LI → LI group. The WT → LI mice exhibited a substantial restoration of synaptic density compared to the LI → LI mice. In addition, the donor microglia-like cells in the WT → LI group showed diminished synaptic protein uptake, indicating a decrease in synaptic elimination (Fig. 5, K and L).

In summary, these findings suggest that neuronal circuit vulnerability to aberrant microglial activity—such as excessive elimination of functional synapses—likely precedes adulthood and may be restricted to early developmental stages, as adult transplantation of *Disc1^LI/LI^* microglia in WT brains did not result in spatial memory deficits. Conversely, replacing the microglia in adult *Disc1^LI/LI^* mice with WT bone marrow-derived cells, which have normal actin cytoskeleton rearrangements, effectively restores synaptic function and reverses deficits in memory consolidation.

## DISCUSSION

The actin cytoskeleton, along with its regulatory proteins, is closely linked to NDDs (*3*–*16*, *20*–*22*). In contrast to the dogmatic neuronal focus of existing research, our study shifts the perspective to explore how the actin cytoskeleton of microglia specifically contributes to cognitive impairments associated with these disorders. We propose a model in which microglial cytoskeletal disorganization, here induced by the deletion of a regulating hub protein, DISC1, impairs phagocytosis, reduces synapse number, and weakens synaptic function within the CA1 region of the hippocampus, ultimately contributing to spatial memory deficits similar to those observed in NDDs including schizophrenia (*36*). These findings offer insight into the role of microglial cytoskeletal regulation in adult brain function, highlighting how deficits in actin remodeling can negatively affect cognition.

While our study emphasizes the cell-intrinsic role of DISC1 in microglia, we fully acknowledge that DISC1 is broadly expressed in the brain and critically regulates neuronal development, signaling, and synaptic function (*37*–*39*). Neuronal dysfunction resulting from DISC1 deficiency is therefore a likely contributor to the overall behavioral phenotype observed in full *Disc1* LI mice. However, our aim was to build upon this established body of work by uncovering additional, microglia-specific roles for DISC1. To isolate these effects, we used a bone marrow transplantation (BMT) strategy in which WT or *Disc1^LI/LI^* hematopoietic precursors repopulate the brain of WT or *Disc1^LI/LI^* hosts. This approach allowed us to dissect the contribution of DISC1 within microglia-like cells in a WT or DISC1-deficient brain environment. A particular feature of our microglia repopulation approach is its ability to reverse specific developmental cognitive deficits in adulthood while leaving motor learning unaffected. This indicates that only certain developmentally miswired connections can be restored by introducing WT microglia-like cells into the adult DISC1-impaired brain, pointing to region-specific microglial activity and/or a differential role of microglia in various neurotransmitter systems. This suggests that microglial DISC1 specifically contributes to neuronal outcomes. Given its high plasticity, the hippocampus is likely particularly reliant on proper microglial function and intact cytoskeletal regulation (*36*). Complementing this, we performed in vitro assays using purified primary microglia in the absence of neurons, confirming that DISC1 regulates microglial behavior—including cytoskeletal organization, motility, and synaptic phagocytosis—in a cell-autonomous manner. These complementary approaches demonstrate that DISC1 deficiency within microglia is sufficient to alter key phagocytic and functional properties, independent of neuronal influence.

In line with the observed cognitive impairments, we sought to better understand how DISC1 regulates microglial phagocytic behavior through cytoskeletal remodeling. Our findings demonstrate that *Disc1^LI/LI^* microglia undergo a shift in cytoskeletal architecture, characterized by a reduced capacity to form ARP2/3-dependent lamellipodia and enhanced formation of formin-driven filopodia. This reorganization is supported by transcriptomic profiling, revealing down-regulation of ARP2/3-complex components and up-regulation of formin-related transcripts, as well as imaging analyses showing increased Formin1 levels upon DISC1 deficiency. These cytoskeletal changes are particularly relevant in the context of phagocytosis, where two actin-dependent engulfment modes have been described in phagocytic cells (*23*). Lamellipodial phagocytosis internalizes larger targets and is ARP2/3-dependent, whereas filopodial phagocytosis enables the engulfment of small or spatially restricted targets via formin-mediated mechanisms. Given that synapses are discrete and microglia operate within spatially constrained brain microenvironments, we propose that microglia may favor filopodial phagocytosis, contributing to excessive uptake of synaptic material upon DISC1 deficiency. This interpretation aligns with our findings of increased filopodia dynamics and enhanced synaptic engulfment in *Disc1^LI/LI^* microglia, providing plausible mechanistic insight into how DISC1-dependent cytoskeletal pathways might influence phagocytic selectivity and efficiency. Moreover, the coexistence of enhanced synaptic engulfment and reduced motility corresponds with the filopodia-rich morphology of *Disc1^LI/LI^* microglia. The loss of lamellipodia likely impairs broad membrane ruffling required for surveillance, whereas the increased number and motility of filopodia may enhance the ability to engulf and internalize synaptic elements. This reasoning supports the finding that DISC1-deficient microglia undergo a functional transition that impairs surveillance yet promotes synaptic elimination.

Although DISC1 mutations are rare and not commonly detected in genome-wide association studies for specifically categorized diagnostic criteria such as schizophrenia, this model has been invaluable for uncovering the mechanisms behind cytoskeletal dysfunction in NDDs in a cross-disease manner. As a scaffold protein, DISC1 interacts with various cytoskeletal regulators of which many are implicated in NDDs, hence coordinating their function in both neurons and microglia (*3*–*16*, *20*–*22*). DISC1 deficiency enables the exploration of cytoskeletal disruptions that extend beyond genetic risk factors associated with common NDD variants. By investigating how microglial cytoskeletal dynamics contribute to synaptic maintenance and plasticity, our findings highlight that targeting microglial cytoskeleton regulation is useful in addressing cognitive impairments linked to NDDs, even in adulthood.

## MATERIALS AND METHODS

### Animals

All animal experiments complied with the European Community guiding principles and were approved by the Ethical Committee on Animal Research of Hasselt University. Animals were group housed (unless otherwise stated) in a temperature (21°C)– and humidity-controlled room with ad libitum access to food and water on a 12-hour light-dark cycle. The *Disc1* LI model was created as described in (*37*–*40*). Homozygous mutated (*Disc1^LI/LI^*) and WT (*Disc1^WT/WT^*) littermates were bred from heterozygous *Disc1* LI mice. A double mutant *Disc1^LI/LI^ Cx3cr1^eGFP/+^* mouse model was created in-house, expressing eGFP under the *Cx3cr1* promoter, marking all monocyte-derived cells, including microglia, as green fluorescent (*41*). *Cx3cr1^eGFP/eGFP^* mice were sourced from the European Mouse Mutant Archive institute with S. Jung’s approval (Weizmann Institute of Science). *Disc1^WT/WT^ Cx3cr1^eGFP/+^* and *Disc1^LI/LI^ Cx3cr1^eGFP/+^* littermates were bred by crossing *Disc1^WT/LI^ Cx3cr1^eGFP/eGFP^* males with *Disc1^WT/LI^ Cx3cr1^+/+^* females. Age-matched mice of either sex were used for all experiments and combined with littermate controls.

### BMT and microglial depletion

Recipient 10-week-old *Disc1^WT/WT^* and *Disc1^LI/LI^* mice were anesthetized intraperitoneally with ketamine (30 mg/kg) and xylazine (5 mg/kg) in sterile phosphate-buffered saline (PBS) before being γ-irradiated with a sublethal dose of 8 grays. Immediately after irradiation, mice received an intravenous injection of 5 × 10^6^ bone marrow cells in sterile PBS from the tibia and femurs of 6-week-old sex-matched *Disc1^WT/WT^ Cx3cr1^eGFP/+^* and *Disc1^LI/LI^ Cx3cr1^eGFP/+^* mice. Bone marrow cells haploinsufficient at the *Cx3cr1* (*Cx3cr1^eGFP/+^*) locus are favored in central nervous system engraftment and generation of microglia-like progeny cells as compared to WT (*Cx3cr1^+/+^*) counterparts (*42*). Mice were given filtered tap water with Neomycin (100 mg/liter; Gibco, USA) and Polymyxin B sulfate (60,000 U/liter; Sigma-Aldrich, USA) for 2 weeks before and 2 weeks after irradiation. One week post-BMT, mice received chow with colony-stimulating factor 1 receptor (CSF1R) inhibitor PLX5622 [standard chow (1200 mg/kg); Chemgood, USA] for 2 weeks (*43*). Experiments began 13 weeks after BMT.

### Behavioral tests

Behavioral tests were conducted every other day during the light phase (50 lux) (unless otherwise stated) (*44*). The experimental timeline progressed from less to more aversive tests, and mice were accustomed to handling.

#### General, instinctive, and repetitive behavioral tests

##### Strength tests

On a grip strength apparatus, an animal grasped a small grid with its front and hind paws while being gently pulled backward until it released the handle (*45*). The maximum force (N) of the animal’s grip was recorded. The latency to fall from an inverted metal cage grid was analyzed as a measure of strength as well (*46*).

##### Marble burying test

Mice were individually placed in standard polycarbonate filtertop cages (*L* × *B* × *H*: 26 cm by 48 cm by 20 cm) filled with 7-cm clean bedding. Twenty standard glass marbles were arranged in a 4 × 5 grid pattern on top of the bedding. Mice were left undisturbed in the test cage for 30 min. A marble was counted as “buried” if at least 75% of it was submerged by bedding.

##### Nest building and nestlet shredding

Mice were individually placed in standard polycarbonate filtertop cages filled with clean bedding and a preweighed cotton fiber nestlet square (5 cm by 5 cm, 5 mm thick; Bio Services, The Netherlands). Mice were left undisturbed for 30 min or 24 hours. The weights of the remaining nonshredded nestlet material were determined after 24 hours of drying to calculate the percentage of nestlet shredded. The nestlets were scored as described in (*47*): (i) nestlet seemed untouched, (ii) nestlet was minimally shredded, (iii) nestlet was mostly shredded without an identifiable nest, (iv) nestlet was mostly shredded and identifiable as a flat nest, and (v) nestlet was mostly or completely shredded and identifiable as a proper nest in the form of a crater.

##### Water spray test

The mouse was put in a plastic test chamber (*L* × *B* × *H*: 38 cm by 38 cm by 50 cm), placed toward the spray nozzle (20 to 30 cm away), and sprayed four times with room-temperature water to adequately coat the mouse’s dorsal surface with mist. The induced self-grooming behavior of the mouse was recorded for 15 min.

#### Anxiety and depression assessments

##### Dark-light shuttle box

The apparatus was a box (*L* × *B* × *H*: 42 cm by 21 cm by 21 cm) divided into a dark and light compartment of equal size. A small opening joined the two compartments, and the light compartment was recorded. A mouse was placed in the light compartment and allowed to move freely for 10 min. The cumulative time spent in the light compartment was analyzed.

##### Elevated zero maze

The apparatus consisted of a black circular arena (50 cm in diameter, 5-cm path width, and 70 cm above floor level). A mouse was placed in the middle of an open arm, and behavior was recorded for 5 min.

##### Open field test

The open field apparatus was a white squared arena of 38.5 cm. A mouse was placed in a corner of the arena, facing the wall, and allowed to roam freely for 20 min. The trajectory was recorded for 20 min via a video camera connected to a video tracking system (EthoVision, Noldus, The Netherlands) (*48*, *49*). The time spent in different areas of the open field (center versus walls + corners) was analyzed in the first 5 min.

##### Tail suspension test

The mouse tail was introduced in a plastic cylinder to avoid tail climbing, and tape was applied to 2 cm of the tail tip, suspending the mouse ~1 m above the floor. Immobility time (none of the four paws moving) was measured during a 6-min trial.

##### Forced swim test

Measuring beakers (*D* × *H*: 14 cm by 18 cm) were filled with tap water (25 ± 1°C) to a depth of 10 cm. Mice were suspended by their tail and carefully placed in the water individually. Their subsequent mobility was recorded for 6 min. Cumulative time spent mobile [i.e., any movements other than those necessary to balance the body and keep the head above the water (*50*)] was analyzed during the last 4 min.

#### Cognitive tests

##### Object location task

Cognitive testing was performed during the dark phase, with individually housed mice given minimal cage enrichment. The object location task (OLT) assesses spatial memory and discrimination as previously described (*51*). The apparatus was a circular polyvinyl chloride arena (40 cm by 40 cm) with a transparent front half and a floor illuminated at 20 lux. Four sets of objects were used: a white cube with rounded edges and a smaller, cylindric-shaped bottom stand (5 cm by 5 cm by 8.5 cm), a solid aluminum cube with a square bottom and tapering top (4.5 cm by 4.5 cm by 8.5 cm), a brass bullet-shaped object (3 cm by 11 cm), and a solid metal cube (5 cm by 3 cm by 7.5 cm) with two holes of 0.5 cm. Mice first explored the empty arena twice and then explored the arena with two objects for 2 days, with objects being immovable and their type and location balanced.

Each testing session included two 4-min trials in the test apparatus with a 3-hour intertrial interval in their home cage. During the learning trial, two identical objects were placed symmetrically on the midline of the arena. In the test trial, one object was moved 10 cm forward or backward. Exploration time was manually recorded, defined as the mouse directing its nose to (< 2-cm distance) or touching the object. Sitting on the object was not considered exploration. Mice with exploration times less than 4 s during the learning trial were excluded. The discrimination index, representing memory performance, was calculated as (time exploring the moved object – time exploring the stationary object)/(total exploration time), providing a relative measure of memory performance.

##### Novel object recognition task

The novel object recognition task assesses recognition memory (*52*). The apparatus, objects, exclusion criteria, and analysis are identical to those in the OLT experiment. The test setup is similar to the OLT with minor adjustments. A testing session consisted of two 4-min trials with a 4-hour interval. In the learning trial, two identical objects were placed symmetrically on the midline of the arena. In the test trial, one object was replaced by a novel object. The discrimination index, representing memory performance, was calculated as (time exploring the novel object – time exploring the familiar object)/(total exploration time).

##### Y-maze

The Y-maze alternation task assesses spatial working memory (*53*, *54*). The apparatus consisted of three enclosed transparent plastic arms set at a 120° angle and spatial cues surrounding the testing apparatus. Mice are placed at the center and allowed to explore for 6 min. An alternation is defined as entries into all three arms consecutively. The maximum alternations equal the total arm entries minus two, and the percentage spontaneous alternation is calculated as (actual alternations/maximum alternations) × 100. A significantly higher percentage than 50% indicates well-functioning working memory.

##### Rotarod motor learning test

To assess the acquisition of skilled behavior in mice, a modified rotarod test was used to emphasize learning (*55*). Mice were placed on a 3-cm-diameter drum of an accelerated rotarod machine (Ugo Basile) with their heads pointing away from the observer. The speed increased from 4 to 60 rpm over 5 min. The latency to fall from the rod was recorded manually. The time after which an animal made three consecutive turns on the rod was also registered as the end time of the trial. To evaluate long-term memory, the test was conducted three times per day with 1-hour intervals for five consecutive days. The average time to fall was plotted per day to assess motor learning skills.

### Electrophysiology

Adult (23-week-old) animals were euthanized by decapitation. Brains were rapidly removed and placed in an ice-cold cutting solution containing 140 mM C_5_H_14_ClNO, 2.5 mM KCl, 1.25 mM NaH_2_PO_4_, 7 mM MgCl_2_, 26 mM NaHCO_3_, 0.5 mM CaCl_2_, and 15 mM d-glucose, equilibrated with a 95% O_2_−5% CO_2_ mixture. Coronal slices, 300 μm thick, were prepared using a vibratome (VT1200S, Leica) and allowed to recover for 1 hour at 34°C in artificial cerebrospinal fluid (aCSF) composed of 120 mM NaCl, 2.5 mM KCl, 1.25 mM NaH_2_PO_4_, 2 mM MgCl_2_, 25 mM NaHCO_3_, 2 mM CaCl_2_, and 10 mM d-glucose. Recordings were conducted on pyramidal neurons in the CA1 of the dorsal hippocampus. During recordings, slices were perfused with aCSF (124 mM NaCl, 2.7 mM KCl, 1.25 mM NaH_2_PO_4_, 1.3 mM MgSO_4_, 26 mM NaHCO_3_, 2 mM CaCl_2_, 18 mM d-glucose, 2 mM HC_6_H_7_O_6_, and 95% O_2_–5% CO_2_) at a rate of 1 to 2 ml/min at 34°C. Whole-cell voltage-clamp recordings were performed using borosilicate-glass pipets (Hilgenberg) with resistance of 5 to 7 megohm, pulled with a micropipette puller (P-1000, Sutter Instruments). sEPSCs and mEPSCs were recorded at a holding potential of −70 mV using a Digidata 1440A amplifier (Axon Instruments, USA) controlled by pCLAMP software (Axon). The intracellular solution contained 120 mM CsMeSO_3_, 1 mM MgCl_2_, 1 mM CaCl_2_, 10 mM CsCl, 10 mM Hepes, 1 mM QX-314, 11 mM EGTA, 2 mM Na_2_–adenosine 5′-triphosphate, and 0.3 mM Na–guanosine 5′-triphosphate (pH 7.3). sEPSCs were recorded in the presence of 10 μM gabazine, 1 μM strychnine, and 0.1 μM dihydro-β-erythroidine to isolate glutamatergic events by minimizing γ-aminobutyric acid–mediated, glycinergic, and cholinergic contributions, respectively. For mEPSCs, 1 μM tetrodotoxin was added to the sEPSC-modified aCSF. Analysis of the synaptic events was performed in the NeuroExpress software. The recordings were split into segments of *X* s before analysis, allowing quantification and visualization of low or zero frequencies in cumulative distribution plots. Mean frequency and amplitudes were quantified for no more than two cells per mouse, ensuring adequate representation of biological variability.

### Human iMicroglia

Fetal human microglia were obtained at a premyelinating gestational age of 14 to 20 weeks, and adult human microglia were sourced from normal-appearing white matter resected during frontal or temporal lobe surgeries for non–tumor-related intractable epilepsy, following published procedures (*56*). iMicroglia precursors (from iPSC line AH016-3 Lenti_RFP_IP) and neurons (from iPSC line SFC856-03-03) were cocultured to obtain differentiated iMicroglia as described in (*57*). For Western blot analysis, red fluorescent protein (RFP)^+^–iMicroglia were sorted from the coculture using fluorescence-activated cell sorting (FACS).

### Primary microglia isolation from mice

Cerebral microglia were isolated as described previously (*58*). Briefly, P1 to P3 mouse brains were dissected. The cerebellum and meninges were removed. Cerebra were disintegrated in Dulbecco’s modified Eagle’s medium (DMEM) with 1% penicillin/streptomycin (P/S). Cell suspensions were filtered (70 μm), centrifuged (5 min at ×500*g*), and resuspended in DMEM with 10% fetal calf serum, 10% horse serum, and 1% P/S. Cells were seeded in poly-d-lysine (PDL; 20 μg/ml)–precoated flasks and incubated at 37°C and 5% CO_2_. After 5 to 7 days, the medium was refreshed and supplemented with 30% CSF1-conditioned medium collected from L929 cells. After 7 to 10 days, primary microglia were separated from the mixed glial culture by orbital shaking (3 hours at 230 rpm at 37°C) and seeded onto PDL-precoated (20 μg/ml) glass coverslips (10^5^ cells per well), 96-well plates (10^5^ cells per well), 24-well plates (250 × 10^3^ cells per well), or 35-mm MatTek glass-bottom dishes (10^5^ cells per dish). Experiments were performed within 3 to 5 days after seeding.

### In vitro phagocytosis assay

The flow cytometric phagocytosis assay was performed as described previously (*59*). Primary microglia were seeded in PDL (20 μg/ml)–precoated 96-well plates and incubated with 1,1′-dioctadecyl-3,3,3′,3′- tetramethylindocarbocyanine perchlorate (DiI)–stained synaptosomes isolated using Syn-PER Synaptic Protein Extraction Reagent (Thermo Fisher Scientific) for 10, 30, 60, or 120 min at 37°C and 5% CO_2_. Cells were then washed, detached, and resuspended in filtered FACS buffer [PBS, 2% fetal bovine serum (FBS), and sodium azide] on ice. Data were acquired using the FACSAria II and analyzed with FACSDiva 6.1.3 software (BD Biosciences). Debris and clumped cells were excluded using forward scatter/side scatter dot plots. At least 10,000 cells were acquired per sample. To distinguish synaptosome internalization from membrane attachment, a control experiment in which cells were incubated with synaptosomes at 4°C was used as a negative control for phagocytosis.

### Immunofluorescence, imaging, and analyses

#### Immunocytochemistry and imaging

Primary microglia were fixed with 4% paraformaldehyde (PFA) for 15 min and washed three times with washing buffer (0.2% Triton X-100 in 1× PBS) for 5 min each. Cells were then incubated with blocking buffer (0.2% Triton X-100, 0.3% Tween 20, and 5% bovine serum albumin) for 1 hour at room temperature. The blocking buffer contained 5% sucrose for F-actin staining. Primary antibodies [β-tubulin (1:1000), St John’s Laboratory; Formin1 (1:300), Bioss Antibodies; and C1q (1:500), Abcam] were incubated overnight at 4°C. The next day, the secondary antibodies (1:1000; Thermo Fisher Scientific), optionally combined with Phalloidin–Alexa Fluor 647 (1:40; Thermo Fisher Scientific), were incubated for 1 hour at room temperature. After washing, cells were incubated with 4′,6-diamidino-2-phenylindole (DAPI; 1:10,000) for 15 min, and mounted onto glass slides with Fluoromount-G (Thermo Fisher Scientific). Three-dimensional (3D) Airyscan images were captured using a Zeiss LSM 880 confocal microscope with a Plan-Apochromat 63x/1.4 Oil differential interference contrast (DIC) M27 oil immersion objective lens. Z-stacks encompassed the entire cell with a *z*-scaling of 250 nm and a pixel size optimized to 47 × 47 nm. The microscope, acquisition configuration, and data processing were managed with ZEN Black 2.6 SP1 software. Image analysis was performed using Fiji and ImageJ plugins, including the actin distribution quantification (*60*).

#### Live-cell F-actin imaging

Primary microglia cultured in glass-bottom dishes (300 × 10^3^ cells per dish), precoated with PDL (20 μg/ml), were transduced with a LifeAct-mScarlet Lentivirus in combination with polybrene (8 μg/ml; Santa Cruz Biotechnology, USA) for 7 to 8 hours and washed afterward. The LifeAct-mScarlet plasmid was provided by H. E. de Vries [Amsterdam University Medical Center (UMC)]. Five days after transduction, cells were rinsed and imaged in Krebs [150 mM NaCl, 6 mM KCl, 10 mM Hepes, 10 mM glucose, 1.5 mM CaCl_2_, and 1 mM MgCl_2_ (pH 7.4)]. Live-cell F-actin images were obtained on the Zeiss Elyra PS.1 with a Plan-Apochromat 63x/1.40 Oil DIC M27 objective [numerical aperture (NA) of 1.40], at 512 × 512 pixels every 6 s for 5 min, followed by SIM processing [imaging and analysis protocols are adapted from (*61*)].

Analysis of F-actin dynamics in filopodia was performed using the PIVlab toolbox in MATLAB release 2021a (9.10.0.1649659) as described before (*61*). The contrast was enhanced using a contrast-limited adaptive histogram equalization (set at 20 pixels) and denoised (set at 3 pixels) using preprocessing built-in options. Particle displacements were quantified using a fast Fourier transform window deformation algorithm in interrogation areas of 32 pixels (pass 1) and 16 pixels (pass 2). Velocity vectors were filtered to exclude low contrast areas, and heatmaps representing velocity magnitudes were generated.

For analysis of F-actin dynamics in lamellipodia, all images were processed using Fiji software as described before (*61**–**64*). Each slice of every stack was filtered (median filter = 1), followed by subtraction of background with a ball size of 30. The 3D stacks were then registered for drifting by applying the StackReg plugin with rigid body transformation (*65*). All lamellipodia regions were manually binarized by applying the Huang threshold. Threshold values were set to ensure the presence of the lamellipodia in all the different frames. To quantify the covered area by a lamellipodium, for each movie, consecutive binarized images were pairwise subtracted to generate a new movie consisting of pixels that represent moving lamellipodia. The covered area was then calculated as the sum of these pixels over a time span of 5 min.

#### Raster image correlation spectroscopy

The pCAGEN backbone (no. 11160, Addgene) was used to generate constructs expressing eGFP fused to the C- or N- terminus of human *DISC1* (NM_001012957) under the CAG promoter using EcoRI/Nhe1 and Nhe1/NotI restriction sites, respectively. The negative control vector contained only the eGFP sequence. Ligations were performed with the rapid DNA dephosphorylation and ligation kit (Roche), and plasmids were isolated with a plasmid DNA purification kit (MACHEREY-NAGEL) according to the manufacturer’s instructions.

Microglial BV2 cells were seeded in Ibidi eight-well chambered #1.5 polymer coverslips at a density of 20,000 cells per well in DMEM supplemented with 10% FBS and 1% P/S (37°C and 5% CO_2_). Cells were magnetofected using Glial-Mag with 250 ng of either pCAGEN (DISC1 or control) plasmid along with 200 ng of mCherry-LifeAct plasmid, according to the manufacturer’s instructions. Before imaging, cells were washed with PBS and incubated with SPY650-tubulin (1:2000; Spirochrome) diluted in phenol red–free OptiMEM for 1 hour.

For imaging, a Zeiss LSM 880 laser scanning microscope with Plan-Apochromat 63x/1.2 Water objective (NA of 1.2) and MBS488/543/633 beam filter was used. eGFP, mCherry-LifeAct, and SPY650-tubulin were excited with 488-nm argon-ion (0.8 μW), 543-nm helium-neon (4 μW), and 633-nm helium-neon (3 μW) lasers. The detection for each of the respective channels was 490 to 543 nm, 579 to 614 nm, and 678 to 730 nm. For each measurement, at least 100 frames were acquired at 4 μm above the membrane of the cells. Images contained 256 × 256 pixels with 50-nm pixel size to have multiple pixels in the point-spread function. Pixel dwell, line, and image times were set to 8.19 μs, 4.92 ms, and 1.26 s, respectively. Detection of fluorescence emission light occurred in photon-counting channel mode. All the measurements were carried out at room temperature. Analyses were performed in the software package PAM as described in (*66*, *67*). Briefly, slow fluctuations, such as cell and cell organelle movement, and spatial inhomogeneities were removed from the raw image data with a high-pass filter. The autocorrelation function was calculated by correlating the different frames using the arbitrary-region RICS algorithm (*66*)

$$G\left(\xi ,\psi \right)=\frac{\langle \delta I_{\text{RICS}}\;\left(x,y\right)\cdot \delta I_{\text{RICS}}\;\left(x+\xi ,y+\psi \right)\rangle }{{\langle I_{\text{RICS}}\rangle }^{2}}$$

where ξ and ψ are the spatial lags in pixels, the · is the correlation operator, the angled brackets represent the average of all included pixels within the cytosolic mask, and *<I_RICS_>* is the average of all moving-average corrected pixels included in the region of interest used for analysis. Hence, the nucleus was excluded from the analysis. Last, the diffusion coefficient was calculated by

$$\begin{array}{c} G\left(\xi ,\psi \right)=\frac{\gamma }{N}\;{\left(1+\frac{4D\;|\xi \tau_{p}+\psi \;\tau_{l}\;|}{\omega_{r}^{2}}\right)}^{-1}\;\text{exp}\left[-\frac{\delta r^{2}\left(\xi^{2}+\psi^{2}\right)}{\omega_{r}^{2}+4D\;|{\xi \tau }_{p}+\psi \;\tau_{l}\;|}\right] \end{array}$$

where γ is the shape factor for a 2D Gaussian and equals 2^–3/2^, and τ_p_ and τ_l_ are pixel and line dwell times, δ_r_ is the pixel size, and ω_r_ is the lateral waist of the focus determined by calibration measurements of Atto 488 diffusing freely in solution at 23°C.

#### Adult acute living brain slices

Mice were euthanized by decapitation, followed by quick brain dissection in oxygenated ice-cold slicing solution containing 120 mM *N*-methyl-d-glucamine, 2.5 mM KCl, 25 mM NaHCO_3_, 1 mM CaCl_2_, 7 mM MgCl_2_, 1.2 mM NaH_2_PO_4_, 20 mM d-glucose, 2.4 mM Na^+^ pyruvate, 1.3 mM Na^+^-l-ascorbate (pH 7.3 to 7.4) at ~300 mosmol. Brains were coronally sliced (300 μm thick) using a vibratome (VT1200S, Leica) and allowed to recover for 1 hour at 36°C in oxygenated aCSF containing 126 mM NaCl, 2.5 mM KCl, 26 mM NaCHO_3_, 2 mM CaCl_2_, 2 mM MgCl_2_, 1.25 mM NaH_2_PO_4_, 10 mM d-glucose (pH 7.3 to 7.4) at ~300 mosmol. Experiments were performed under continuous perfusion of oxygenated (95% O_2_ and 5% CO_2_) aCSF at room temperature to preserve slice health. Acute brain slice imaging was performed using a Zeiss LSM 880-NLO-Airyscan confocal microscope (LD C-Apochromat 40x/1.10 W Korr M27) provided with a Mai Tai DeepSee Ti:Sapphire-pulsed laser tuned at 920 nm (13-mW intensity and 1.54-μs pixel dwell). Stacks were recorded starting from a minimum depth of 50 μm above the surface of the slice to avoid cells being activated by slicing (*68*, *69*). A z-stack spanning 14 μm with a 1-μm depth interval and serial optical sections at 512 × 512 pixels (125.03 μm by 125.03 μm) was acquired every min for 10 min. Each line was scanned four times with a depth of 16 bits, and line averaging was used to improve the signal-to-noise ratio of the images. For imaging, the laser power was adjusted to ~13 mW.

Images were processed using Fiji software as described before (*61*). For motility index quantification, the number of new pixels (additions) at each time point was normalized to the total number of pixels in the cells (cell size), therefore giving a quantification of motility normalized to cell size. The extension and retraction speed of microglial processes were calculated by tracking individual branches using the MTrackJ plugin (*70*) for a total duration of 10 min.

#### Fixed brain slices

##### Colocalization and phagocytosis analysis

Three hundred–micrometer–thick free-floating sections were prepared as described for electrophysiological recordings and fixed overnight in 4% PFA. Antigen retrieval was performed in 10 mM citrate buffer (pH 8.5) at 80°C for 30 min. After rinsing in PBS, sections were blocked in 5% normal donkey serum and 5% normal goat serum for 1 hour and then incubated overnight with primary antibodies: rabbit anti-PSD95 (1:500; Alomone Labs) and mouse anti-vesicular glutamate transporter 1 (vGLUT1, 1:2000; Merck Millipore). The next day, sections were rinsed, stained with secondary antibodies [goat anti-mouse A555 (1:500) and goat anti-rabbit A647 (1:500), Thermo Fisher Scientific], counterstained with DAPI, and mounted. Images were captured in Airyscan mode on a Zeiss LSM 880-NLO-Airyscan confocal microscope (AxioObserver Z.1, Zeiss) with a Plan-Apochromat 63x/1.40 Oil DIC M27 objective. Excitation of Alexa Fluor 488, 555, and 647 was achieved using argon-ion (488 nm), He-Ne (543 nm), and He-Ne (647 nm) lasers, respectively. Image processing was conducted using Fiji software (ImageJ). Colocalization was defined as a distance of less than 50 nm between channels after applying a 1.10% threshold (*71*). Synaptic engulfment was identified when puncta were entirely within the microglial cell body.

##### Microglial morphology

Mice were anesthetized by intraperitoneal injection of 2.5 mg/g (body weight) of Dolethal (Virbac AH) and transcardially perfused with cold PBS containing heparin [20 IU; Heparin LEO (5000 IU/ml)], followed by 4% cold PFA. Brains were dissected and incubated in 4% PFA overnight at 4°C, washed with PBS, and kept in PBS-azide (0.01%) until slicing. Free-floating sections (100 μm) were cut using a Microm HM 650V Vibratome and stained with DAPI for 15 min. The sections were mounted on microscope slides (Thermo Fisher Scientific) and coverslipped in fluorescent Immu-Mount medium. Cortical sections were imaged using a confocal microscope (Zeiss LSM 880-NLO-Airyscan), and eGFP^+^ microglia or microglia-like cells infiltrated after BMT were visualized using the Argon 488-nm laser. Microglial cells were imaged within a 20-μm z-stack (slice thickness of 1 μm) at 3136 × 3136 pixels (267.18 μm by 267.18 μm) using a Plan-Apochromat 63x/1.40 Oil DIC M27 objective.

Microglial morphology was assessed by Sholl analysis as described in (*61*, *62*, *72*). Briefly, cell reconstructions were performed using 3D automatic cell tracing in Vaa3D software (www.vaa3D.org) using the APP2 (All-path-pruning 2.0) algorithm to generate 3D skeletons of the ramified microglia (*73*). The morphological features were analyzed using a length-based hierarchical pruning method, as previously described by Kyrargyri *et al*. (*62*). Custom codes in MATLAB are available at https://github.com/AttwellLab/Microglia.

### Western blotting

Freshly isolated cells were lysed on ice in cold radioimmunoprecipitation assay lysis buffer containing 50 mM tris, 150 mM NaCl, 1 mM EDTA, 1% IGEPAL CA-630, and 0.50% Na^+^ deoxycholate supplemented with protease inhibitor (Roche). Protein concentrations were determined using the bicinchoninic acid protein assay kit (Thermo Fisher Scientific). Equal protein amounts (10 μg of protein, diluted in Laemmli buffer with 5% β-mercaptoethanol and heated for 4 min at 95°C) were used for SDS–polyacrylamide gel electrophoresis (SDS-PAGE; 12% SDS-PAGE gel). Gels were transferred to a polyvinylidene difluoride membrane and blocked for 2 hours with a blocking buffer (tris-buffered saline with 0.1% Tween-20 containing 5% skimmed milk). The custom mouse monoclonal 2B3 anti-DISC1 antibody raised against 594 to 852 amino acids of mouse DISC1 [1:500; (*39*)] was subsequently incubated overnight at 4°C, followed by washing steps and incubation with a horseradish peroxidase–conjugated secondary antibody for 1 hour. All antibodies were diluted in blocking buffer, and incubations were at room temperature unless stated otherwise. Proteins were visualized using the enhanced chemiluminescence system (Pierce ECL Western Blotting Substrate, Thermo Fisher Scientific) and ImageQuant LAS 4000. Homogenous loading was checked using β-actin (Sigma-Aldrich). Quantification was performed using Fiji software (*63*).

### Quantitative PCR

Cells were lysed using QIAzol reagent (QIAGEN), and mRNA was isolated using the RNeasy mini kit (QIAGEN), according to the manufacturer’s instructions. cDNA was synthesized using the qScript cDNA synthesis kit (Quanta Biosciences), according to the manufacturer’s instructions. Primer sequences (5′-3′) against mouse transcripts are *C1qa*, CTGAAGATGTCTGCCGAGCA [forward (FW)], and *C1qa*, GCTCCTGGCTCCCCTCTC [reverse (REV)]; *C1qb*, GGATAAAGGGGGAGAAAGGGCT (FW), and *C1qb*, TTAGGGCCAACTTTGCCTGGAG (REV); *C1qc*, CCAAGGGAGAGCCAGGAATC (FW), and *C1qc*, ATTTTTCCCACGGTGGCCA (REV); *Cyca* (housekeeping gene), GCGTCTCCTTCGAGCTGTT (FW), and *Cyca*, AAGTCACCACCCTGGCA (REV); and *Tbp* (housekeeping gene), ATGGTGTGCACAGGAGCCAAG (FW), and *Tbp*, TCATAGCTACTGAACTGCTG (REV). qPCR was performed on a QuantStudio3 detection system (Applied Biosystems). Data were analyzed using the ΔΔCt method.

### Single-cell RNA-seq

Processing and tissue collection were performed as previously described (*74*), using the Act-Seq method (*75*) to limit dissociation-induced gene expression. Briefly, the brains of 23-week-old homozygous mutated (*Disc1^LI/LI^*) and WT (*Disc1^WT/WT^*) littermate controls (*n* = 6 per group) were pooled, cut into small pieces with a scalpel, and then incubated with enzyme mix [deoxyribonuclease I (30 U/ml), Roche; collagenase type I (10 U/ml; Worthington), and collagenase type IV (400 U/ml; Worthington) diluted in 1× Hanks’ balanced salt solution (HBSS, Gibco)], containing 15 μM Actinomycin D at 11°C for 40 min. Every 10 min, the solution was cut and resuspended to ensure full dissociation of the tissue. Subsequently, the solution was resuspended, filtered twice over a 100-μm nylon filter, and centrifuged at 400*g* for 10 min. The pellet was resuspended in 5 ml of 70% standard isotonic Percoll (SIP; GE Healthcare), diluted in 1× HBSS, and gently overlaid with 5 ml of 30% SIP, followed by a 5-ml layer of 30% SIP, forming a three-layered density gradient (centrifuged at 650*g* at 4°C for 30 min without acceleration or braking). All gradient buffers contained 3 μM ActD. The cell cloud at the 70/30% interphase was collected, centrifuged, and resuspended in staining buffer (2 mM EDTA and 2% FBS in 1× HBSS). Subsequently, cells were incubated with anti-mouse CD16/CD32 (clone 2.4G2), anti-CD45 Pacific Blue (clone 30-F11, BioLegend), and CD11bAF647 (clone M1/70, BioLegend) for 20 min on ice. CD45^low^CD11b^+^ immune cells were sorted into 100% FBS using a BD FACSARIA II with a sorting nozzle of 85 μm. Cellular suspensions were loaded on a Chromium Chip B (10x Genomics, no.1000074) or Chip G (10x Genomics, no. 2000177) on a GemCode Single Cell Instrument (10x Genomics) to generate single-cell gel beads-in-emulsion (GEM). GEMs and scRNA-seq libraries were prepared using the GemCode Single Cell 3′ Gel Bead and Library Kit (v3 and v3.1, 10x Genomics, no. 1000075) and the Chromium i7 Multiplex Kit (10x Genomics, no. 120262) according to the manufacturer’s instructions.

Sequencing libraries were loaded on an Illumina HiSeq 4000 flow cell with sequencing settings following the recommendations of 10x Genomics. The Cell Ranger pipeline (10x Genomics) was used to perform sample demultiplexing and to generate FASTQ files for read 0, read 2, and the i7 sample index. Read 2, containing the cDNA, was mapped to the reference genome (mouse mm10) using STAR. Subsequent barcode processing, unique molecular identifiers filtering, and single-cell 3′ gene counting were performed using the Cell Ranger suite and Seurat v.3.0.1. The total number of cells across all libraries was 8112 cells. The filtered matrices were processed using the Seurat integration workflow (*76*). The SCTransform function was used to normalize counts based on read depth, and the 2000 most variable features per sample were identified for integration with corrected counts. Integration anchors between matrices were identified using reciprocal principal components analysis (PCA), and genes for integration were ranked by their presence in the matrices. Subsequent dimensionality reduction used preprocessed genes and values, while raw and normalized counts were maintained for subsequent testing for differential expression. Dimensionality reduction was performed using PCA, resulting in 25 principal components. An elbow plot confirmed that these 25 principal components contained the major sources of variation in the dataset. These components were then used as input for uniform manifold approximation and projection (UMAP) dimensionality reduction. For each identified cluster, DEGs were calculated by comparing gene expression within the cluster to all other clusters. The statistical workflow for differential expression followed Seurat’s implementation of the Wilcoxon signed-rank test. DEGs were deemed significant if the Bonferroni-adjusted *P* value was less than 0.05 and the natural log fold change was below −0.25 or above 0.25. To investigate the differential regulation of biological pathways in each cluster, DEGs exceeding the significance threshold were exported to IPA (Qiagen) for a comprehensive core and comparative analysis.

### Statistical analysis

Statistical analysis and graphs were produced using Prism 9 (GraphPad Software, USA). Data distributions were assessed for normality (Shapiro-Wilk), and parametric or nonparametric two-tailed tests were applied accordingly. Nonparametric tests were applied when the data distribution of one or more groups was non-Gaussian. Figure legends include details on sample sizes, statistical analysis, and representation. *P* values smaller than 0.05 were considered significant.
