# Supplementary material for: Cytoskeletal control in adult microglia is essential to restore neurodevelopmental synaptic and cognitive deficits
Source: Sci Adv. 2025 Aug 29;11(35):eadw0128. doi: 10.1126/sciadv.adw0128 (PMC12396340; doi:10.1126/sciadv.adw0128)
Supplement: Supplementary file 1 — Figs. S1 to S6 [file sciadv.adw0128_sm.pdf]

Science Advances
AAAS

Supplementary Materials for

Cytoskeletal control in adult microglia is essential to restore neurodevelopmental synaptic and cognitive deficits

Sofie Kessels *et al.*

Corresponding author: Bert Brône, bert.brone@uhasselt.be; Akira Sawa, asawa1@jhmi.edu

Sci. Adv. **11**, eadw0128 (2025)
DOI: 10.1126/sciadv.adw0128

This PDF file includes:

Figs. S1 to S6

Supplementary Figures and Figure Captions (Fig. S1 – Fig. S6)

FIGURE NUMBER	TITLE	Page
**FIGURE S1**	Characterization of clusters in scRNAseq analysis.	2
**FIGURE S2**	Gene expression patterns associated with the complement cascade, phagosome formation, and actin cytoskeleton signaling across distinct microglial clusters.	3
**FIGURE S3**	*Cd55* is abundant in a specific group of microglia absent in *Disc1* LI mice.	4
**FIGURE S4**	Microglial surveillance is impaired in *Disc1* LI microglia *in vitro*.	5
**FIGURE S5**	Behavioral phenotype of constitutive and chimeric *Disc1* locus impairment mice.	6-7
**FIGURE S6**	*S*ynaptic transmission to dorsal hippocampal pyramidal neurons is impaired in *Disc1* locus impairment mice.	8-9

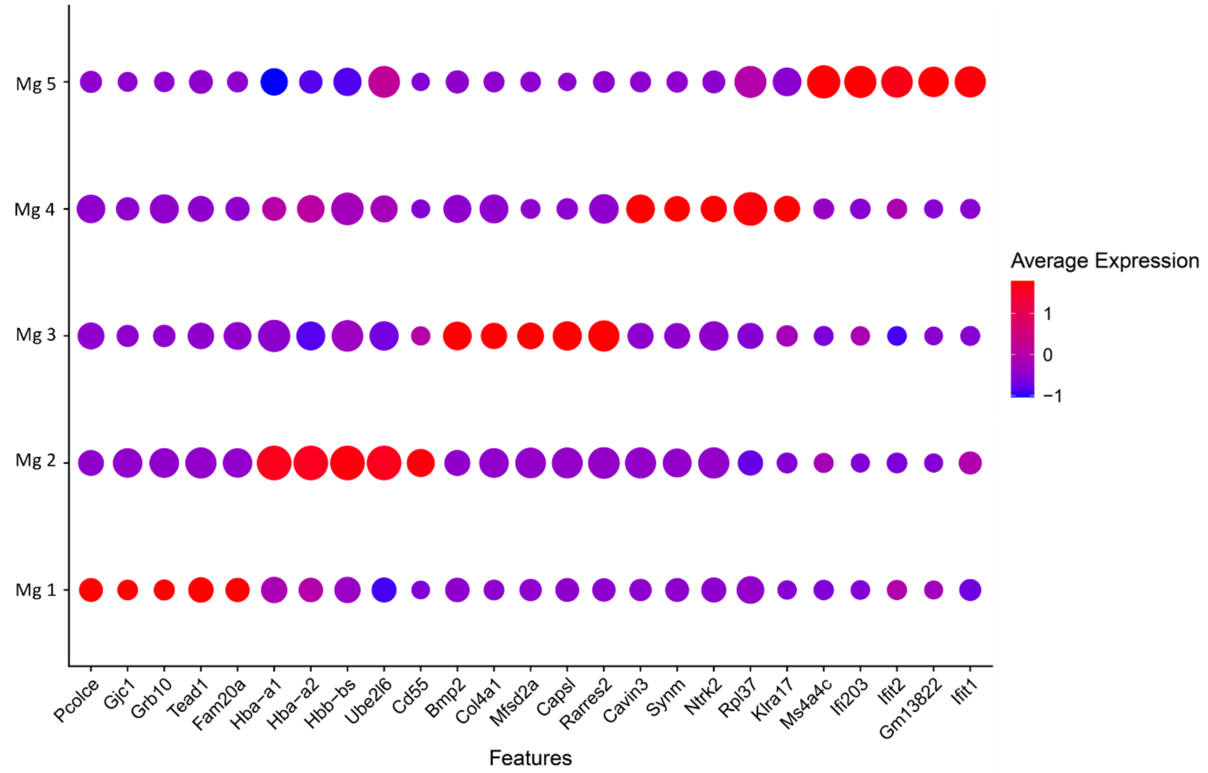

FIGURE S1 | Characterization of clusters in scRNAseq analysis. Microglial clusters are displayed in rows, with columns representing the most differently expressed genes for each cluster. Dot size indicates the fraction of cells in a cluster where the gene was detected, and the dot color reflects the mean expression *z*-score for the cells belonging to that cluster.

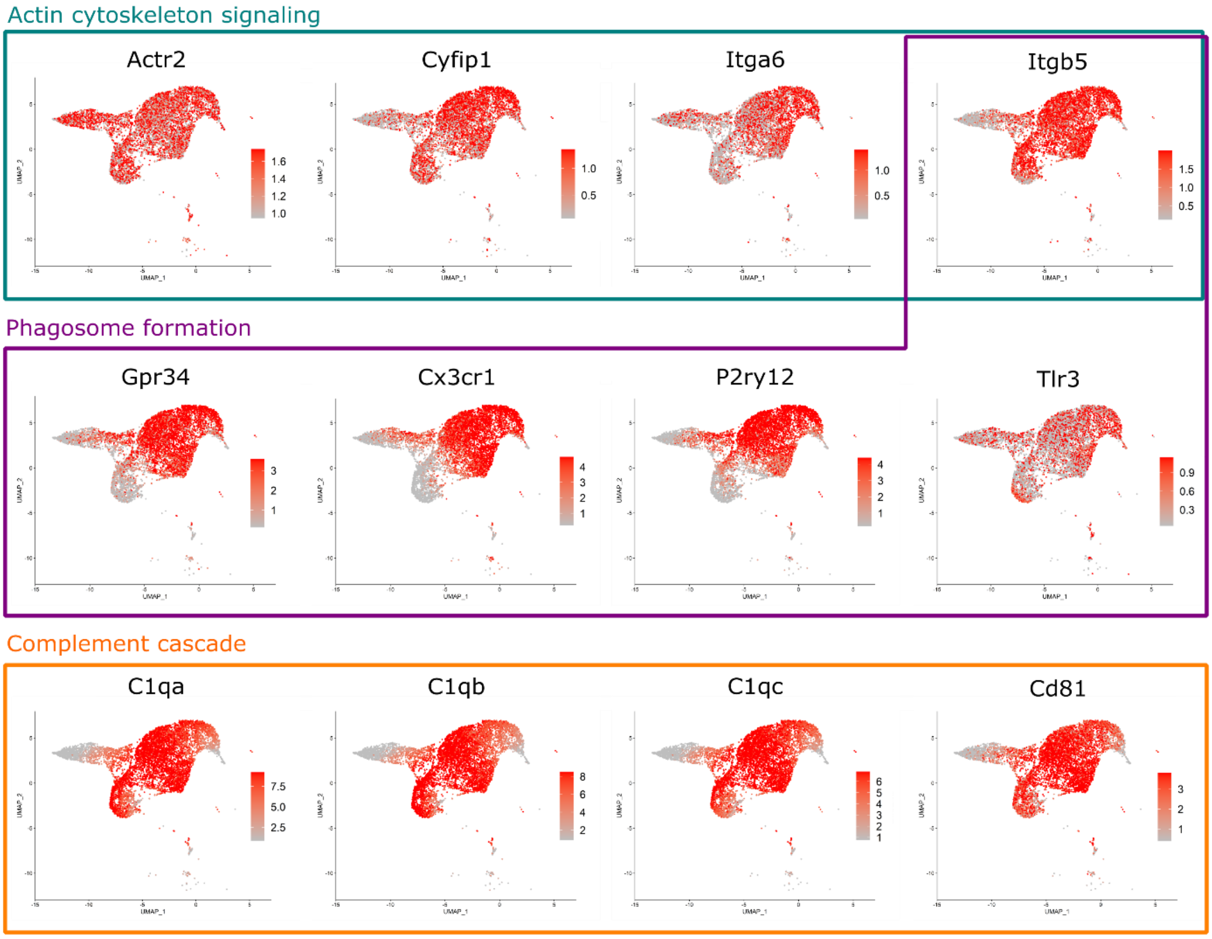

FIGURE S2 | Gene expression patterns associated with the complement cascade, phagosome formation, and actin cytoskeleton signaling across distinct microglial clusters. UMAP visualization of genes enriched in pathways related to actin cytoskeleton signaling, phagosome formation, and complement cascade shows their distribution across all clusters.

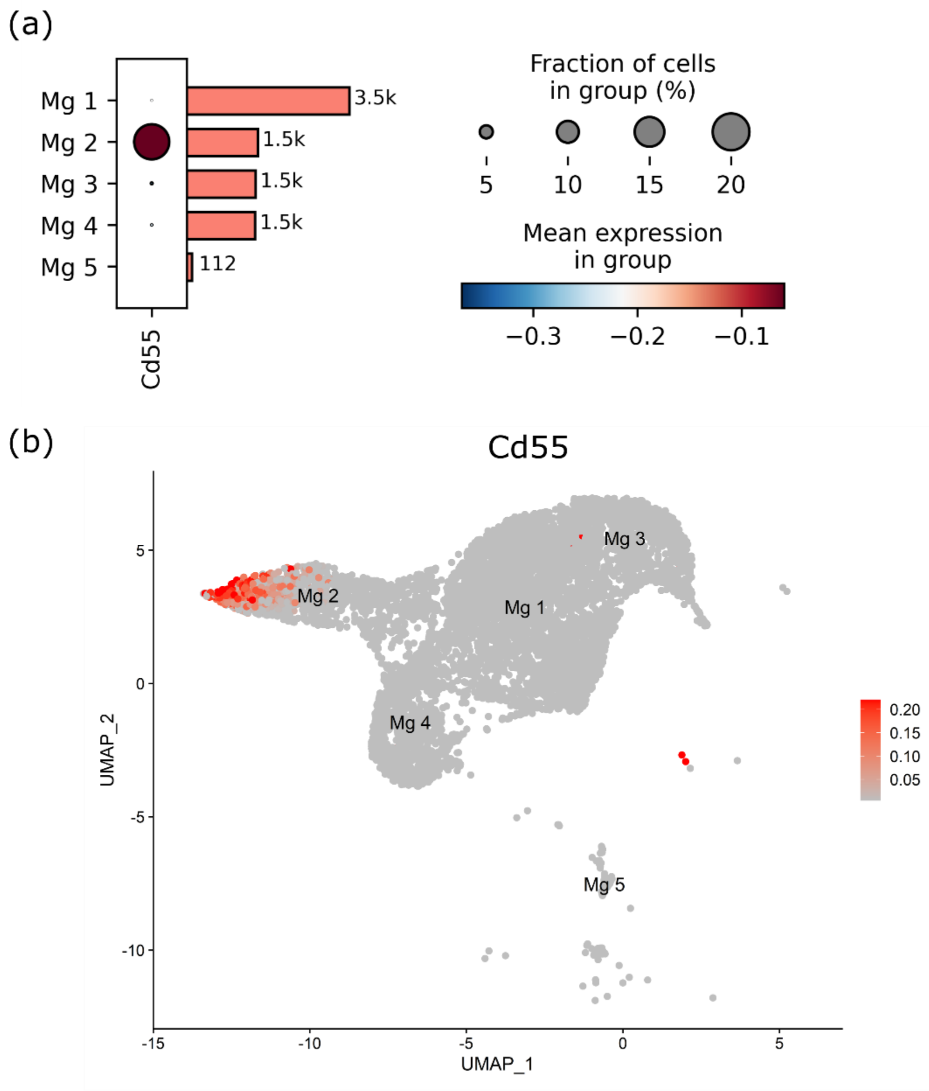

FIGURE S3 | *Cd55* is abundant in a specific group of microglia absent in *Disc1* LI mice. (a) A distribution plot illustrates the high expression of *Cd55* by the Mg2 cluster. The size of each dot corresponds to the fraction of cells in a given cluster where the gene was detected, and the color of the dot represents the mean expression *z*-score for the cells belonging to that cluster. **(b)** UMAP visualization of *Cd55* distribution across all clusters.

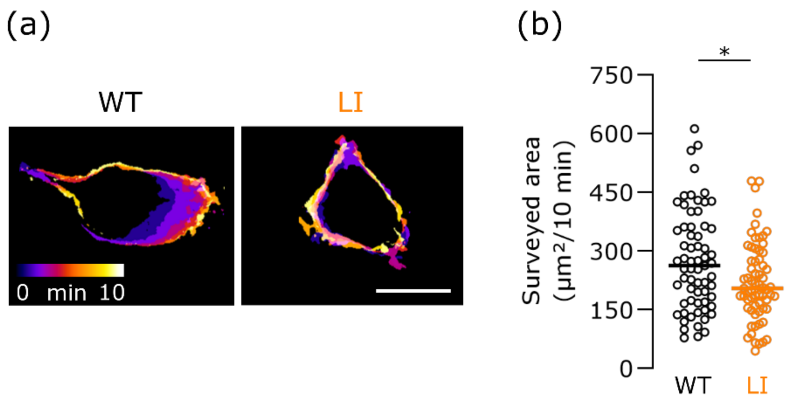

FIGURE S4 | Microglial surveillance is impaired in *Disc1* LI microglia *in vitro*. (a) Representative images of cultured *Disc1*$^{WT/WT}$ and *Disc1*$^{LI/LI}$ microglia incubated with a CellMask plasma membrane stain. Images taken 1 minute apart are overlaid with different colors. Scale bar, 10 µm. **(b)** Total surveyed area in 10 min of *Disc1*$^{WT/WT}$ and *Disc1*$^{LI/LI}$ primary microglia (P = 0.0190, n = 65 per genotype). Data points represent individual microglia. Horizontal bars indicate the median. Shapiro-Wilk test followed by the two-tailed Mann-Whitney U test. * $P < 0.05$

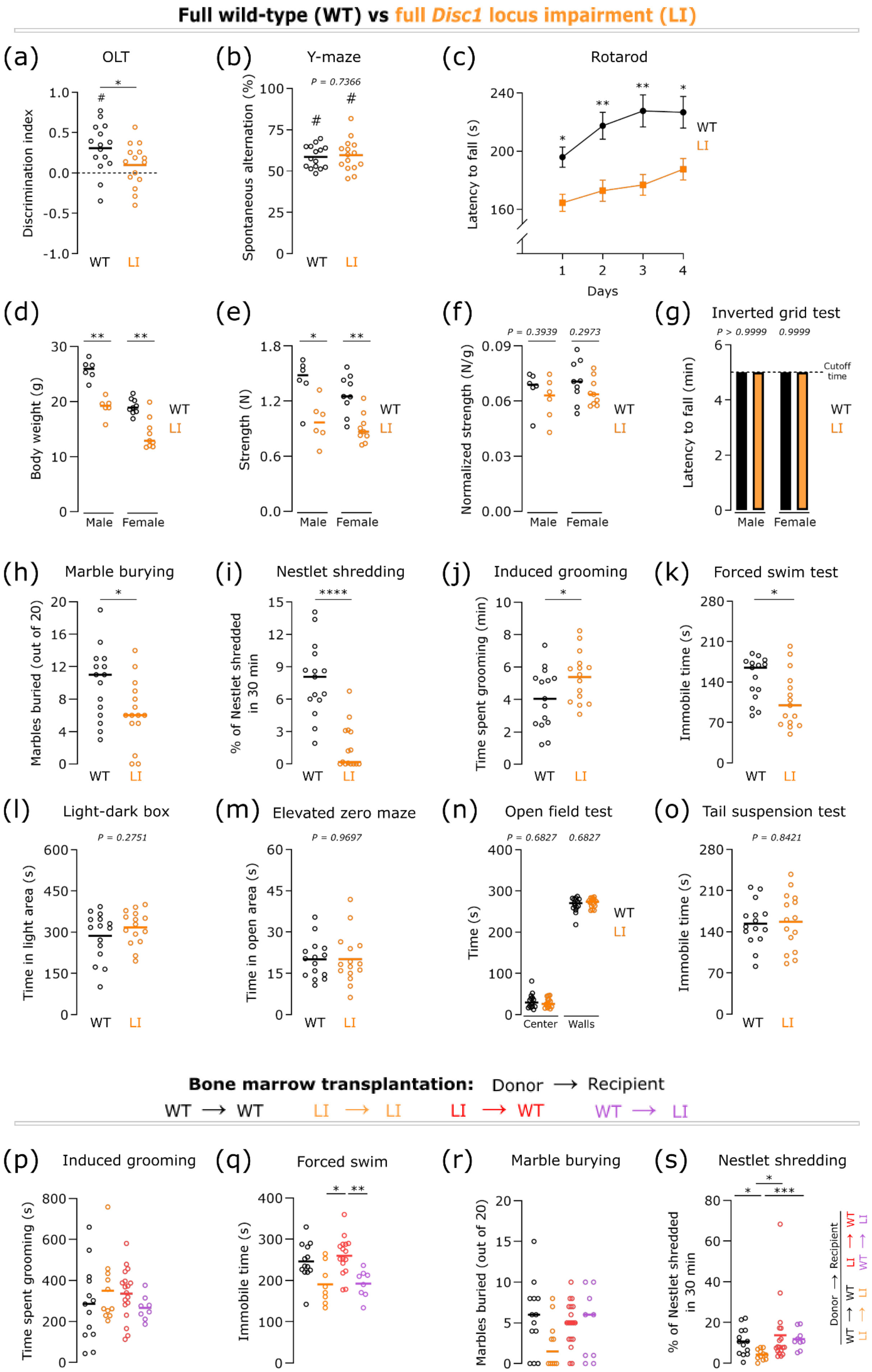
Full wild-type (WT) vs full Disc1 locus impairment (LI)
(a) OLT
Discrimination index
WT LI
(b) Y-maze
P = 0.7366
Spontaneous alternation (%)
WT LI
(c) Rotarod
Latency to fall (s)
Days
WT
LI
(d)
Body weight (g)
Male Female
WT
LI
(e)
Strength (N)
Male Female
WT
LI
(f)
P = 0.3939 0.2973
Normalized strength (N/g)
Male Female
WT
LI
(g) Inverted grid test
P > 0.9999 0.9999
Cutoff time
Latency to fall (min)
Male Female
WT
LI
(h) Marble burying
Marbles buried (out of 20)
WT LI
(i) Nestlet shredding
% of Nestlet shredded in 30 min
WT LI
(j) Induced grooming
Time spent grooming (min)
WT LI
(k) Forced swim test
Immobile time (s)
WT LI
(l) Light-dark box
P = 0.2751
Time in light area (s)
WT LI
(m) Elevated zero maze
P = 0.9697
Time in open area (s)
WT LI
(n) Open field test
P = 0.6827 0.6827
Time (s)
Center Walls
WT
LI
(o) Tail suspension test
P = 0.8421
Immobile time (s)
WT LI
Bone marrow transplantation: Donor → Recipient
WT → WT
LI → LI
LI → WT
WT → LI
(p) Induced grooming
Time spent grooming (s)
(q) Forced swim
Immobile time (s)
(r) Marble burying
Marbles buried (out of 20)
(s) Nestlet shredding
% of Nestlet shredded in 30 min
Donor → Recipient

FIGURE S5 | Behavioral phenotype of constitutive and chimeric *Disc1* locus impairment mice. **(a)** Object location test (P = 0.0408) (# = significantly different from zero in the one-sample t-test). **(b)** Y-maze alternations (# = significantly different from 50% in the one-sample t-test). **(c)** Latency to fall from the rotarod during the first day of motor learning (P = 0.0267), and a steeper learning curve for $Disc1^{WT/WT}$ *than for* $Disc1^{LI/LI}$ mice (P = 0.0033, 0.0030, and 0.0267 on day 2, 3, and 4, respectively). Data points represent mean ± SEM. Two-way repeated measures ANOVA with Sidak's multiple comparisons test. **(d)** Body weight (male: P = 0.002165, female: P = 0.001851). **(e)** Total muscle strength (male: P = 0.015152, female: P = 0.001234). **(f)** Strength normalized to body weight. **(g)** Inverted grid test. **(h)** Marble burying test (P = 0.0457). **(i)** Nestlet shredding test (P < 0.0001). **(j)** Water spray-induced grooming (P = 0.0396). **(k)** Immobility duration in the forced swim test (P = 0.0262). No difference between genotypes in **(l)** the light-dark transition box, **(m)** the elevated zero maze, **(n)** the open field test, **(o)** the tail suspension test, **(p)** water spray-induced grooming. **(q)** The elicited immobility duration in the forced swim test is dependent on the acceptor's *Disc1* genotype. **(r)** No difference in marble burying between groups. **(s)** Nestlet shredding is significantly higher for all groups compared to LI→LI mice. **(a-s)** Data points represent individual animals unless stated otherwise. **(a-b, k-m, o)** Horizontal bars indicate the mean. Shapiro-Wilk test followed by an unpaired two-tailed t-test. **(d-j, n)** Horizontal bars indicate the median. Shapiro-Wilk test followed by multiple two-tailed Mann-Whitney *U* test. **(p-s)** Horizontal bars indicate the median. Kruskal-Wallis with Dunn's multiple comparison test. * P < 0.05, ** P < 0.01, *** P < 0.001, **** P < 0.0001

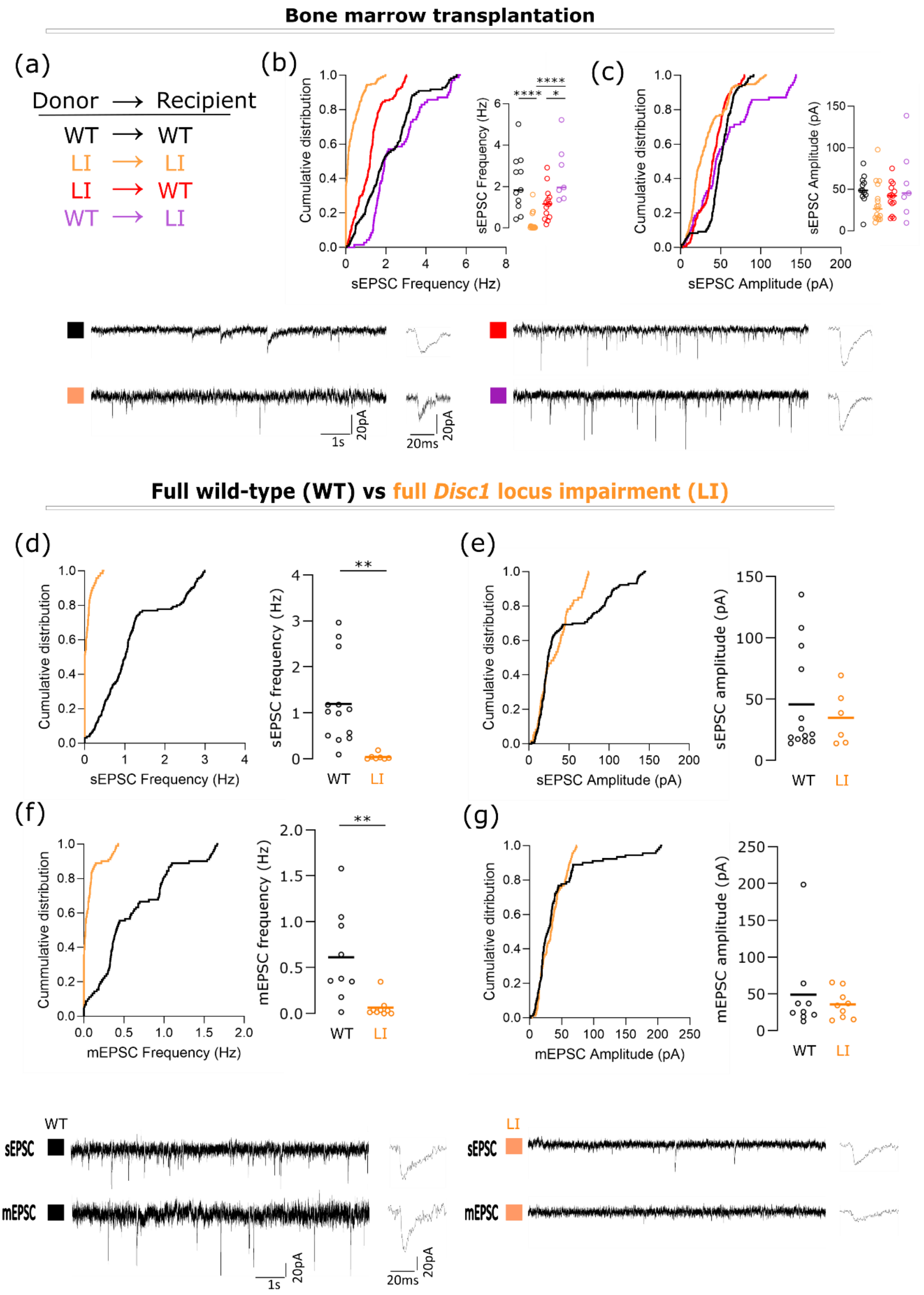
Bone marrow transplantation
(a)
Donor → Recipient
WT → WT
LI → LI
LI → WT
WT → LI
(b)
Cumulative distribution
sEPSC Frequency (Hz)
****
****
*
(c)
sEPSC Amplitude (pA)
1s
20pA
20ms
Full wild-type (WT) vs full Disc1 locus impairment (LI)
(d)
sEPSC frequency (Hz)
**
WT
LI
(e)
sEPSC amplitude (pA)
(f)
Cummulative distribution
mEPSC Frequency (Hz)
mEPSC frequency (Hz)
(g)
Cumulative ditribution
mEPSC Amplitude (pA)
mEPSC amplitude (pA)
sEPSC
mEPSC

FIGURE S6 | Synaptic transmission to dorsal hippocampal pyramidal neurons is impaired in *Disc1* locus impairment mice. (a) Illustration of utilized chimeric groups. Representative current recordings of sEPSCs with an enlarged view of a single synaptic event per condition are shown. Cumulative distribution and quantitative analysis of **(b)** the frequency and **(c)** the amplitude of sEPSC of dorsal pyramidal CA1 neurons clamped at -70 mV suggests significantly decreased neuronal transmission in the dorsal hippocampus of LI → LI mice compared to WT → WT mice (Cum Prob P = <0.0001; Insert P = <0.0001) and compared to *Disc1*$^{LI/LI}$ mice receiving *Disc1*$^{WT/WT}$ bone marrow (WT→LI) (Cum Prob P = <0.0001; Insert P = 0.0006). One-way ANOVA with Tukey's multiple comparisons test (n = 7-17, 5-6 mice). In 23-week-old full *Disc1*$^{LI/LI}$ mice, cumulative probabilities and quantitative analysis of **(d)** the frequency of sEPSC and **(f)** mEPSC of dorsal pyramidal CA1 neurons clamped at -70 mV shows decreased hippocampal transmission (sEPSC: Cum Prob P = <0.0001, Insert P = 0.0042; mEPSC: Cum Prob P = 0.0006, Insert = P 0.0052). (n = 7-17 cells; 5-6 mice). Cumulative probabilities and quantitative analysis of **(e)** the amplitude of sEPSC and **(g)** mEPSC measurements of dorsal pyramidal CA1 neurons from 23-week-old full *Disc1*$^{LI/LI}$ mice clamped at -70 mV show no significant differences (n = 6-13, 5-6 mice). **(b-g)** Data points represent individual cells. Horizontal bars indicate the median. (d-g) Student's *t*-tests. * P < 0.05, ** P < 0.01, **** P < 0.0001
